# Membrane architecture and adherens junctions contribute to strong Notch pathway activation

**DOI:** 10.1242/dev.199831

**Published:** 2021-10-14

**Authors:** Julia Falo-Sanjuan, Sarah J. Bray

**Affiliations:** Department of Physiology, Development and Neuroscience, University of Cambridge, Downing Street, Cambridge CB2 3DY, UK

**Keywords:** Adherens junctions, MS2 transcription, Notch, Cellularization, *Drosophila*

## Abstract

The Notch pathway mediates cell-to-cell communication in a variety of tissues, developmental stages and organisms. Pathway activation relies on the interaction between transmembrane ligands and receptors on adjacent cells. As such, pathway activity could be influenced by the size, composition or dynamics of contacts between membranes. The initiation of Notch signalling in the *Drosophila* embryo occurs during cellularization, when lateral cell membranes and adherens junctions are first being deposited, allowing us to investigate the importance of membrane architecture and specific junctional domains for signalling. By measuring Notch-dependent transcription in live embryos, we established that it initiates while lateral membranes are growing and that signalling onset correlates with a specific phase in their formation. However, the length of the lateral membranes per se was not limiting. Rather, the adherens junctions, which assemble concurrently with membrane deposition, contributed to the high levels of signalling required for transcription, as indicated by the consequences of α-Catenin depletion. Together, these results demonstrate that the establishment of lateral membrane contacts can be limiting for Notch trans-activation and suggest that adherens junctions play an important role in modulating Notch activity.

## Abstract (precis)

**Summary:** Measuring Notch-dependent transcription in live embryos reveals that features associated with lateral membranes are required for initiation of Notch signalling. Perturbing membrane growth or adherens junctions prevents normal activation.

## INTRODUCTION

The Notch pathway is a cell-cell signalling pathway that is conserved across animals and has widespread roles in development, homeostasis and disease. Following interactions between the Notch receptor and its transmembrane ligands of the Delta or Serrate/Jagged families in adjacent cells, Notch is cleaved and the intracellular domain (NICD) translocates to the nucleus, where it regulates transcription of target genes. As ligands are transmembrane proteins (except for some examples in *C. elegans*; D'Souza et al., 2008; Chen and Greenwald, 2004), signalling is limited to cells that are directly in contact, although in some cases the contacts may occur through long cellular processes that extend a considerable distance (Hunter et al., 2019; Boukhatmi et al., 2020; Cohen et al., 2010; Hamada et al., 2014; Nelson et al., 2013). Tissue geometry and the nature of the cell contacts will thus impact on the levels as well as the duration of signal that a cell receives from its neighbours (Shaya et al., 2017). Elucidating the contributions from tissue architecture to Notch signalling will therefore be important to understand how signalling is effectively deployed in the different processes it controls.

An example where the acquisition of cell architecture may be important is during cellularization in *Drosophila*. Profound morphological changes take place at this stage, which corresponds to the onset of Notch signalling in the mesectoderm: a stripe of cells located between the mesoderm and ectoderm that gives rise to the future midline of the ventral nerve cord (Nambu et al., 1990; Morel and Schweisguth, 2000; Morel et al., 2003). Prior to nuclear cycle 14 (nc14), the *Drosophila* embryo is a syncytium – the nuclei divide but are not separated by membranes. During nc14, membranes ingress to build intracellular membranes surrounding each nucleus, creating ∼6000 cells, a process referred to as cellularization (Foe and Alberts, 1983; Lecuit and Wieschaus, 2000; Lecuit et al., 2002).

In analysing the real-time response of two well-characterized Notch responsive mesectodermal enhancers – *m5/m8* from *E(spl)-C* and the mesectodermal enhancer from *single-minded* (*sim*) (Martin-Bermudo et al., 1995; Cowden and Levine, 2002; Zinzen et al., 2006; Hong et al., 2013) during nc14, we observed that Notch-dependent transcription was first detectable 30 min into nc14 (Falo-Sanjuan et al., 2019). This differs from other enhancers active at this stage, which exhibit high levels of activity from the beginning of nc14 (Garcia et al., 2013; Bothma et al., 2014, 2015; Lim et al., 2017). Ectopic production of NICD, which does not depend on membrane release and trafficking, from the beginning of nc14 was sufficient to produce earlier *m5/m8* and *sim* activity, suggesting that factors downstream of NICD production, such as co-activators or chromatin landscape, are not limiting when transcription normally initiates. Based on the fact that, under normal conditions, the two Notch responsive enhancers have similar onset times, we hypothesized that signalling is normally initiated at that time, leading to a sharp release of NICD to initiate transcription (Falo-Sanjuan et al., 2019). Key components required for ligand endocytosis and Notch activation, including the E3 ligase Neuralized, are produced at this stage (Price et al., 1993; Morel et al., 2003; De Renzis et al., 2006) and likely delimit when signalling can be initiated. However, it is difficult to envisage how their expression could result in the tightly synchronized transcription onset times that were observed. An alternate possibility is that additional, highly coordinated morphological events, such as the formation of lateral membranes and cell junctions or the alterations in nuclear morphology (Brandt et al., 2006; Pilot et al., 2006), are involved in gating signalling activity.

The timing and progression of cellularization is coordinated by two zygotically expressed proteins, Slam and Nullo, which are localized to the basal domain of the ingressing membranes (Hunter and Wieschaus, 2000; Lecuit et al., 2002; Postner and Wieschaus, 1994; Rose and Wieschaus, 1992; Wieschaus and Sweeton, 1988; Simpson, 1990; Acharya et al., 2014). Slam activates Rho signalling by recruiting RhoGEF2 to the prospective basal domain, where it promotes actin polymerization and actomyosin contractility, resulting in furrow invagination (Wenzl et al., 2010). Likewise, Nullo stabilizes the lateral furrows by regulating endocytic dynamics, which helps localize proteins to the basal junctions and impacts on actomyosin contractility (Sokac and Wieschaus, 2008a,b). As cellularization proceeds, cadherin-catenin complexes are assembled into first basal and then apical adherens junctions (AJs) that delimit the apical and basolateral domains (Hunter and Wieschaus, 2000; Kramer, 2000). This step-wise progression of lateral membrane growth and junction formation offers a unique opportunity to explore the relationship between lateral membrane growth and competence for Notch signalling. We hypothesized that Notch signalling cannot initiate until the appropriate membrane domains are formed and matured, so that the ligand and receptor can be appropriately juxtaposed. However, it has also been proposed that cis-activation can occur, whereby productive interactions take place between the ligand and receptor in the same cell, either on the cell surface or on intracellular membrane vesicles (e.g. endosomes) (Coumailleau et al., 2009; Nandagopal et al., 2019). It has also been suggested that signalling initiates gradually from the beginning of nc14, before cellularization starts (Viswanathan et al., 2019), which might imply a cis-activation mechanism. By investigating the onset of signalling during cellularization, we aim to resolve these models.

To distinguish the different models to explain signalling onset, we have assessed which processes during cellularization can affect the timing and/or levels of Notch-dependent transcription in the mesectoderm. We find that Notch and Delta are present on the ingrowing lateral membranes and that signalling onset is highly correlated with membrane growth, but not with nuclear shape changes. Furthermore, the results suggest that the presence of lateral membranes per se is not sufficient for activation, and that high levels of signalling also require the establishment of cellular junctions, the integrity of which regulates the turnover of Notch at the membrane. Whether the junctions contribute directly or indirectly to the signalling capabilities remains to be established, but the evidence clearly points to membrane morphogenesis, and the establishment of signalling-competent membrane domains, as a key determinant for the initiation of Notch signalling in the embryo.

## RESULTS

### Initiation of Notch-Delta signalling coincides with growth of lateral membranes

**Fig. 1.**
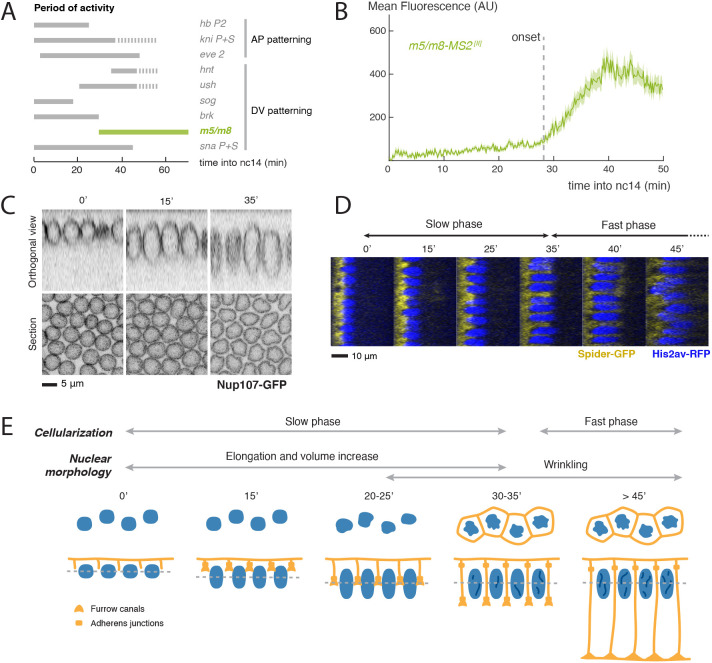
**Correlation between developmental processes and onset of Notch-dependent transcription.** (A) Summary of the expression timings of other published MS2 lines. Grey solid lines indicate activity and dashed lines indicate periods when transcription was not quantified but the enhancer/gene is expected to be active. Based on data from Garcia et al., 2013; Bothma et al., 2014; Bothma et al., 2015; Lim et al., 2017; Falo-Sanjuan et al., 2019; Hoppe et al., 2020. (B) Mean profile of transcription from *m5/m8*. Transcription starts from 30 min into nc14. Based on data from Falo-Sanjuan et al., 2019. (C) Medial sections and orthogonal views of nc14 embryos at the indicated times (min into nc14) expressing the nuclear membrane marker Nup107-GFP. See also Movie 1. (D) Orthogonal views of embryos expressing the cell membrane marker Spider-GFP and nuclei marker His2Av-RFP, indicating changes in nuclear and membrane length over time (min into nc14). (E) Summary of the behaviours of nuclei and membranes during nc14. Cellularization takes place in two phases: slow (0-35′) and fast (35′ onwards) membrane in-growth. At the same time, nuclei elongate and increase in volume (0-35′), and their surface becomes wrinkled from ∼25 min onwards. Embryos were at imaged at 20-22°C.

Notch-dependent transcription in the mesectoderm is first detected ∼30-35 min after the mitosis that marks the start of nc14, as illustrated by activity of *m5/m8* enhancer (Zinzen et al., 2006; Falo-Sanjuan et al., 2019). Expression from this enhancer initiates sharply at this time and rapidly achieves high levels of activity. Its behaviour differs from that of other enhancers at this stage, which are active from the start of nc14 (Fig. 1A,B). High levels of NICD can bypass the temporal restriction, directing much earlier expression of both *m5/m8* and another Notch-regulated enhancer (Falo-Sanjuan et al., 2019), arguing that they are competent to respond in early nc14 and that another step, besides enhancer accessibility, is limiting transcription onset. Nuclear maturation and cellularization are two developmental processes that occur during nc14 and could potentially govern the onset of Notch-dependent transcription in a direct or indirect manner. We therefore began by characterizing how each of these processes related to the timing of transcription, measured using the MS2/MCP system (Garcia et al., 2013) to detect activity from the Notch-dependent *m5/m8* enhancer in real time.

The substantial changes in nuclear morphology that occur during nc14 include volume and shape changes, and alterations in pore clustering (Brandt et al., 2006; Pilot et al., 2006; Hampoelz et al., 2016) that could affect the entry of transcription factors (e.g. Twist) that prime the enhancers. To quantify these nuclear changes, embryos expressing Nup107-GFP (Katsani et al., 2008) were imaged live and the nuclear dimensions and eccentricity measured over time. Nuclei underwent substantial elongation in the apico-basal axis and increased in volume during the first 40 min of nc14 (Brandt et al., 2006; Pilot et al., 2006) (Fig. 1C, Fig. S1A, Movie 1). In addition, after ∼25 min into nc14, there was an increase in eccentricity of nuclear medial slices, indicative of indentations (‘wrinkles’) appearing in the nuclear envelope (Fig. 1C, Fig. S1A, Movie 1) (Brandt et al., 2006; Pilot et al., 2006). This transition to ‘wrinkling’ occurred around the time when signalling-dependent transcription is initiated.

**Fig. 2.**
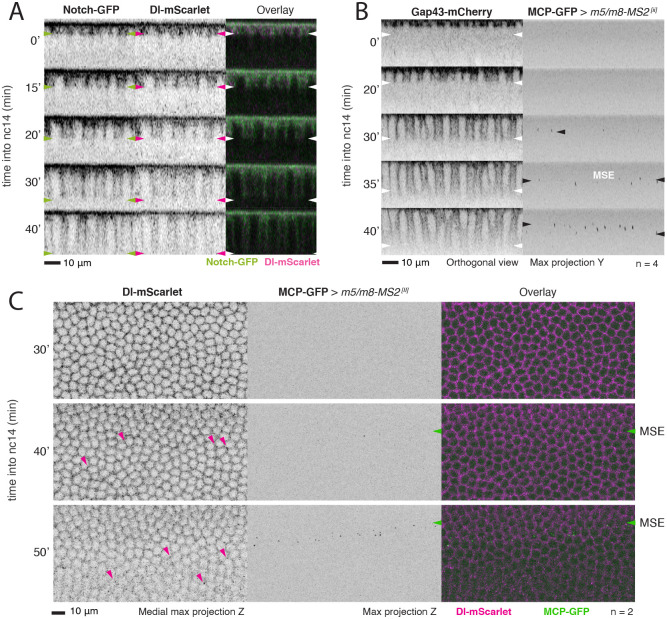
**Notch-responsive transcription starts before cellularization is completed.** (A) Orthogonal views of embryos expressing Notch-GFP and Dl-mScarlet, showing localization of Notch and Delta at cellularizing membranes. Arrowheads indicate the position of the most basal accumulation. See also Movie 2. (B) Stills of a movie of an embryo expressing the membrane marker Gap43-mCherry combined with MCP-GFP to image transcription from *m5/m8*. Orthogonal views of Gap43-mCherry (left) and maximum projections of the MCP-GFP channel (right) are shown. Time into nc14 (min) is indicated for each. Transcription starts from 30 min into nc14 and is visible in the whole mesectoderm stripe by 35-40 min, before cellularization has completed. White arrowheads indicate the position of cellularization front; black arrowheads indicate transcription in mesectoderm nuclei (MSE). See also Movie 3. (C) Stills of an embryo expressing Dl-mScarlet combined with MCP-GFP to image transcription from *m5/m8*. Projections of medial slices (left, inverted image), maximum projections of the MCP-GFP channel (centre, inverted image) and overlay of both (right) at three time points as indicated. Delta can be detected in bright puncta (magenta arrowheads) close to the membrane in mesoderm cells from the time *m5/m8* transcription starts in the mesectoderm (MSE, green arrowheads).

Similarly, we used the membrane marker Spider-GFP (Gilgamesh, Morin et al., 2001) to track the inward-growing, lateral, membranes during cellularization and to quantify their growth. In agreement with previous reports, we could detect an initial slow phase of membrane ingrowth, which lasted ∼30-35 min, followed by a fast phase, where the membranes extended more rapidly to complete cellularization (Foe and Alberts, 1983; Lecuit and Wieschaus, 2000; Lecuit et al., 2002) (Fig. 1D, Fig. S1B). By the end of the slow phase, membranes had reached the inferior margin of the nucleus (Fig. 1D). This corresponded approximately to the time at which Notch-dependent transcription usually initiates (Falo-Sanjuan et al., 2019). Furthermore, these ingrowing lateral membranes carried Notch and Delta. Tracking GFP-tagged Notch, produced from a genomic construct expressing at normal levels (Notch-GFP, Couturier et al., 2012) and endogenously-tagged Delta (Dl-mScarlet, Boukhatmi et al., 2020) revealed that the location of both proteins expanded basally at the same rate as cellularization progressed (Fig. 2A, Fig. S2A), although there may be some apical bias in their distribution, and that Dl-mScarlet tracked with E-cadherin (Shg-GFP) (Fig. S2B,C). Thus, lateral membranes containing Notch and Delta have partially formed at the time when signalling commences.

To relate to the time when Notch-dependent transcription initiates with lateral membrane growth in real time, we monitored transcription directed by the *m5/m8* enhancer in the presence of the membrane marker Gap43-mCherry (Fabrowski et al., 2013), which was used to track lateral membrane growth in cells within and close to the MSE. Results revealed that the onset of *m5/m8*-dependent transcription occurred when the membranes had grown, on average, ∼20* *μm (Fig. 2B, Movie 3). At a similar stage, membrane levels of Delta became modulated in the mesodermal cells, where it was primarily detected in bright puncta close to the membrane. These changes in Dl localization occurred throughout the mesoderm, but not in the mesectodermal cells, where *m5/m8* transcription was initiated (Fig. 2C), and likely correspond to increased Delta endocytosis driven by Neuralized, as reported previously (Morel et al., 2003; De Renzis et al., 2006).

Based on the onset of the transcriptional read-out, these data indicate that productive Notch-Delta signalling is initiated after lateral membranes have started to form, during the transition between the slow and fast phases of membrane elongation and significantly before cellularization finishes. This also corresponds to the period when the nuclei are undergoing morphological changes associated with the maturation of nuclear membranes and pores (Fig. 1E).

### Lateral membranes are limiting for Notch signalling

**Fig. 3.**
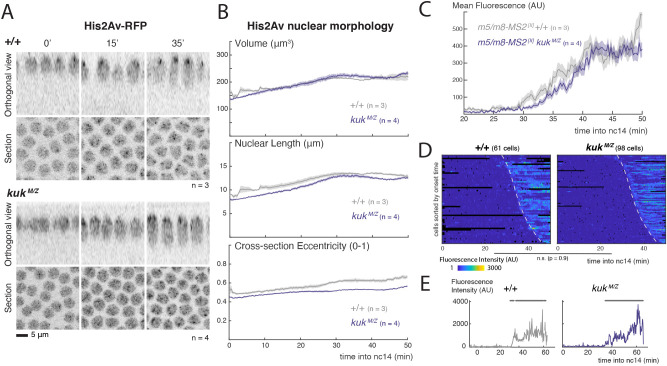
**Changes in nuclear morphology do not influence Notch-dependent transcription.** (A) Cross-sections and orthogonal views of the nuclear marker His2Av-RFP in wild-type embryos (top) and embryos obtained from homozygous *kuk* parents (*kuk^M/Z^*, bottom) at the indicated times (min into nc14). (B) Quantification of nuclear morphological properties over time using the His2Av channel from MS2 experiments: volume, nuclear length and eccentricity of the medial slice. Data are mean±s.e.m. (shaded area) of the properties calculated for each embryo (*n*=number of embryos per indicated condition). (C) Mean profiles of *m5/m8^[II]^* activity of mesectoderm nuclei in control and *kuk* embryos. Data are mean±s.e.m. (shaded area) of all cells combined from multiple embryos (*n*=number of embryos). (D) Heatmaps of transcription in all mesectoderm nuclei sorted by onset time. Dashed lines indicate onset times in the control. n.s., not significant, *P*-value calculated using Kolmogorov–Smirnov test. (E) Examples of transcription traces from mesectoderm nuclei. Horizontal grey lines indicate ON periods.

To distinguish the contributions from nuclear morphogenesis and lateral membrane formation on Notch signalling, we used mutations to perturb each process. First, we investigated the consequences from disrupting nuclear shape changes. *kugelkern* (*kuk*) encodes a nuclear lamina protein required for nuclear elongation and wrinkling at nc14 (Pilot et al., 2006; Brandt et al., 2006). To produce mutant embryos in the context of our experimental assays, we used *kuk[EY07696]*, a characterized allele that has reduced Kuk levels but a milder phenotype than a null allele (Pilot et al., 2006). In agreement with previous studies, nuclei in maternal and zygotic *kuk[EY07696]* mutant embryos (*kuk^M/Z^*) had significantly reduced eccentricity, correlating with a reduction in their indentations (Fig. 3A,B, Fig. S3A), while the overall nuclear volume was unaffected. Transcription directed by *m5/m8* was unaltered in *kuk[EY07696]* mutant embryos; mean levels, onset and transcription profiles were similar to controls (Fig. 3C-E, Fig. S3B). These data suggest that the stage-specific change in nuclear membrane wrinkling is not required for the normal onset and levels of Notch-dependent transcription. However we cannot rule out the possibility that there could be more subtle changes in the nuclear membrane, such as in the functional organization of nuclear pores, that could have a role.

**Fig. 4.**
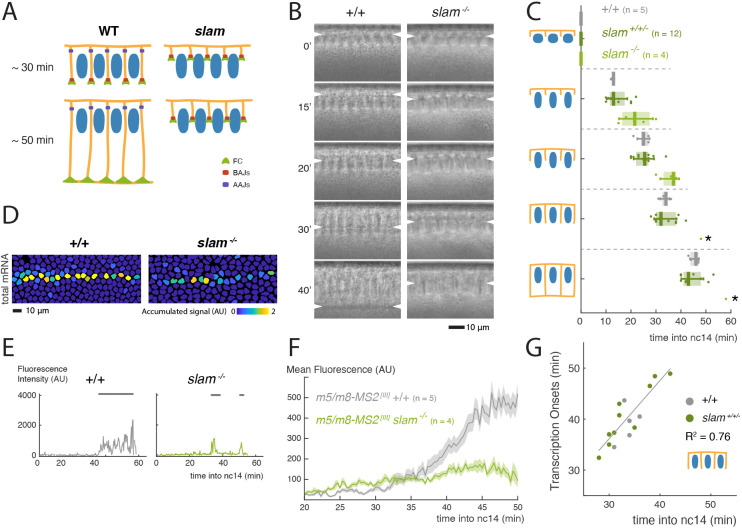
**Lateral membranes are required for Notch signalling.** (A) Schematic representation of the effects on membrane formation produced by mutation of *slam*. (B) Cross-sections of wild-type and *slam^−/−^* embryos captured with transmitted light and used to measure cellularization progression. Arrowheads indicate the position of the cellularization front. (C) Boxplots indicating timing of cellularization progression (timepoints when membranes reach each of the lengths with respect to nuclei indicated in the cartoons) in wild-type, *slam^+/+/−^* and *slam^−/−^* embryos. Median, Q1/Q3 quartiles and s.d. are shown. Asterisks indicate timepoints for only one *slam^−/−^* embryo, as the others arrested during cellularization. (D) Tracked mesectoderm nuclei colour-coded for their total *m5/m8^[III]^* transcription levels. (E) Examples of *m5/m8^[III]^* transcription traces from mesectoderm nuclei. Horizontal grey lines indicate ON periods. (F) Profile of mean *m5/m8^[III]^* activity in *slam^−/−^* embryos compared with controls. (G) Correlation between the timepoint of cellularization when membranes reach the basal end of nuclei with onset of transcription from *m5/m8* (calculated as the first quartile of onset times) in *slam^+/+/−^* and control embryos. R^2^ coefficients are calculated after pooling all points together; correlations for each genotype separately are in Fig. S4. In F, data are mean±s.e.m. (shaded area) of all cells combined from multiple embryos (*n*=number of embryos). See also Movie 4.

Second, we asked whether the formation of lateral membranes is a limiting factor in pathway activation, by analysing the consequences on *m5/m8* transcription from a mutation in the zygotic gene *slam*, which disrupts cellularization (Lecuit et al., 2002). Membrane formation was quantified by capturing a cross-section of the embryo in every time-point using transmitted light (Fig. 4B), and measuring the length of lateral membranes to determine time points when the cellularization front reached specific positions (Fig. 1E) (Lecuit and Wieschaus, 2000; Lecuit et al., 2002; Acharya et al., 2014). In this way, we obtained a read-out for the overall cellularization speed in an individual embryo but could not specifically quantify membrane progression in the MSE where signalling is occurring. This analysis confirmed that cellularization was blocked in homozygous mutant embryos for *slam*: all phases of cellularization were slowed down and it was fully arrested in three out of four embryos (Fig. 4B,C). Strikingly, mesectoderm nuclei exhibited almost no *m5/m8* transcriptional activity in homozygous *slam* mutant embryos. A few nuclei initiated sporadic transcription at the same time as in control embryos, but this lasted only few minutes (Fig. 4D,E, Fig. S4A,B, Movie 4). As a result, mean levels of *m5/m8* transcription were close to background (Fig. 4F). These data argue that, in contrast to nuclear morphogenesis, normal lateral membrane formation is important for signalling to initiate and be maintained.

Live imaging was performed on all of the progeny from the genetic cross and it was notable that a significant proportion of the embryos that were not homozygous *slam* mutants also displayed abnormal cellularization. In these, likely *slam^+/−^* heterozygous, embryos, lateral membrane growth was significantly slowed (Fig. 4C). Because we could not definitively distinguish the *slam^+/−^* heterozygous embryos from any pseudo-normal homozygous balancer embryos, we quantified transcription onset and cellularization times in all non *slam^−/−^* embryos in an unbiased way. The results revealed a striking relationship between cellularization time points and the onset of *m5/m8* activity (first quartile of onset times), with the strongest correlation with the time when membranes reached the basal end of nuclei (*R*^2^=0.76) (Fig. 4G, Fig. S4C,F). Delta localization appeared normal in *slam^−/−^* mutant embryos until lateral membrane growth was arrested or delayed (Fig. S4D,E). Delta thus occupies the available lateral membrane territory in each condition.

Together, these observations indicate that Delta-Notch signalling initiates after the lateral membranes have partially formed but before cellularization finishes. The correlation between onset of transcription and membrane progression suggests that a specific step during cellularization determines when signalling can start. One possibility is that the membrane length per se is limiting because it determines the amount of Notch and Delta that are available for signalling. Alternatively, the formation of a specific membrane domain or junction may be the limiting factor that enables productive Notch-Delta interactions.

### Adherens junctions contribute to Notch activation

**Fig. 5.**
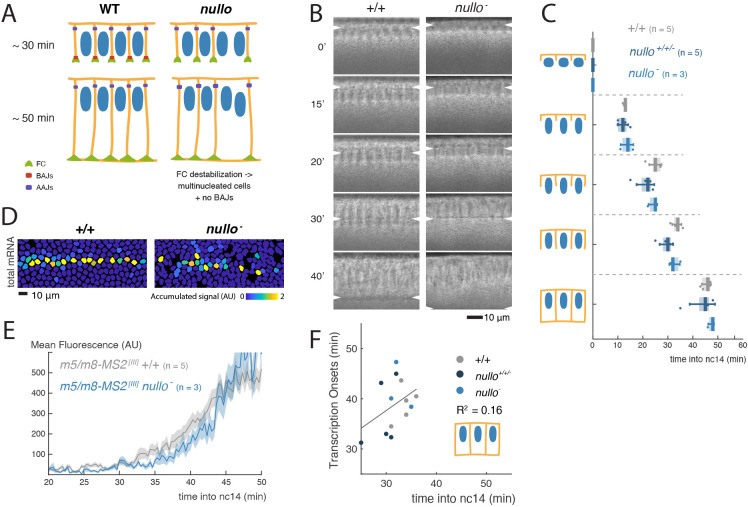
**Defects in cellularization from absence of Nullo perturb Notch signalling independently of membrane growth.** (A) Schematic representation of the effects on membrane formation produced by mutations in *nullo*. (B) Cross-sections of wild-type and *nullo^−^* embryos captured with transmitted light and used to measure cellularization progression. Arrowheads indicate position of the cellularization front. (C) Boxplots indicating timing of cellularization progression (timepoints when membranes reach each of the lengths with respect to nuclei indicated in the cartoons) in wild-type, *nullo^+/+/−^* and *nullo^−^* embryos. Median, Q1/Q3 quartiles and s.d. are shown. (D) Tracked mesectoderm nuclei colour-coded for their total *m5/m8^[III]^* transcription levels. (E) Profile of mean *m5/m8^[III]^* activity in *nullo^−^* embryos compared with control embryos. (F) Correlation between the timepoint of cellularization when membranes reach the basal end of nuclei with onset of transcription from *m5/m8^[III]^* (calculated as the first quartile of onset times) in *nullo^+/+/−^* and control embryos. R^2^ coefficients are calculated after pooling all points together; correlations for each genotype separately are in Fig. S5. Images, plots and quantifications of control embryos are duplicated from Fig. 4. See also Movie 4.

To investigate whether the onset of signalling is limited by the dimensions of the lateral membrane per se or by the establishment of specific domains, such as AJs, we first examined the *m5/m8* transcriptional profiles in embryos mutant for *nullo*, in which furrow canal components are mislocalized and furrow canals are destabilized. In these embryos, the majority of lateral membranes are formed but the transient basal adherens junctions (BAJs) are perturbed (Postner and Wieschaus, 1994; Hunter and Wieschaus, 2000; Hunter et al., 2002). As the apical adherens junctions (AAJs) are subsequently established normally (Hunter and Wieschaus, 2000; Hunter et al., 2002) (Fig. 5A), *nullo* mutants would distinguish whether the BAJs are required.

Overall, the cellularization fronts in *nullo* hemizygous embryos progressed at a similar mean rate to control embryos (Fig. 5B,C), indicating there was not a global defect in lateral membrane growth during the early stages of cellularization. Similarly, the overall mean transcription levels and onset times for *m5/m8* in *nullo* hemizygous embryos resembled those of control embryos, suggesting that BAJs are not essential for Notch activity (Fig. 5E, Fig. S5A-C, Movie 4). We note that a few nuclei failed to initiate transcription, giving rise to a more disorganized and patchy stripe of mesectodermal *m5/m8* activity (Fig. 5D), which could be due to altered signalling in the absence of neighbouring cell membranes (some multinucleate cells were visible at later time points). However, because we were unable to visualize membranes simultaneously with the MS2 system, we did not have the single-cell precision to directly confirm this hypothesis.

Although the overall mean transcription levels and onset times in *nullo* hemizygous embryos were similar to wild type (Fig. 5E, Fig. S5A,B), on an embryo-by-embryo basis there was more variability in the transcription onset times of *nullo* mutant embryos than for controls. We therefore made a comparison between the transcription onset times and the cellularization times for each embryo, as we had done for the *slam* mutants. In contrast to those embryos, there was no correlation between transcription onset times and cellularization in *nullo^+/+/−^*, *nullo^−^* embryos when all were considered together (Fig. 5F, Fig. S5D,E). This suggests that, although lateral membrane growth is important, the overall lateral membrane length is not the limiting parameter for initiation of Notch signalling, and that other consequences from removing Nullo are responsible for the increased variability in onset times.

**Fig. 6.**
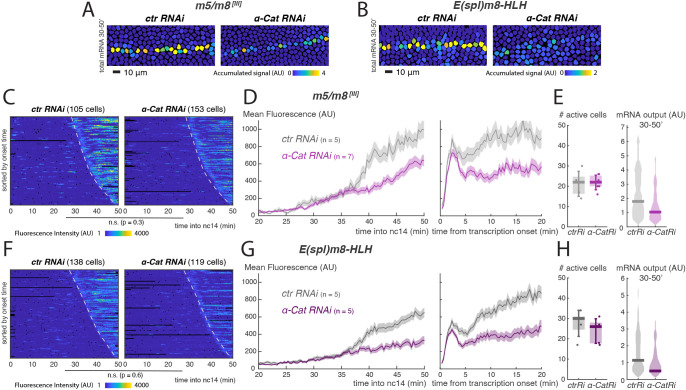
**Adherens junctions influence Notch-dependent transcription.** (A,B) Tracked mesectoderm nuclei colour-coded for total *m5/m8^[III]^* (A) or *E(spl)m8-HLH* (B) transcription (accumulated signal from 30 to 50 min into nc14). (C) Heatmaps of *m5/m8^[III]^* transcription in all mesectoderm nuclei sorted by onset time in control and α-Cat-depleted embryos. (D) Profile of mean *m5/m8^[III]^* activity in *α-Cat RNAi* embryos compared with controls, aligned by developmental time (left) or transcription onset times (right). (E) Boxplot indicating number of mesectoderm cells transcribing *m5/m8^[III]^* in each embryo in control and *α-Cat RNAi* embryos (left: median, Q1/Q3 quartiles and s.d. are shown) and violin plot showing the distribution of output levels of transcription (accumulated signal from 30 to 50 min into nc14), as in A, combining all nuclei from each condition (right: distribution and median shown). (F) Heatmaps of *E(spl)m8-HLH* transcription in all mesectoderm nuclei sorted by onset time in control and α-Cat-depleted embryos. (G) Profile of mean *E(spl)m8-HLH* activity in *α-Cat RNAi* embryos compared with controls, aligned by developmental time (left) or transcription onset times (right). (H) Boxplot indicating number of mesectoderm cells transcribing *E(spl)m8-HLH* in each embryo in control and *α-Cat RNAi* embryos (left: median, Q1/Q3 quartiles and s.d. are shown) and violin plot showing the distribution of output levels of transcription (accumulated signal from 30 to 50 min into nc14), as in B, combining all nuclei from each condition (right: distribution and median shown). In D and G, data are mean±s.e.m. (shaded area) of all cells combined from multiple embryos (*n*=number of embryos). In C and F, dashed lines indicate onset times in controls. n.s., not significant, *P*-value calculated using Kolmogorov–Smirnov test. See also Movies 5 and 6.

The results suggest that features associated with the lateral membranes are required for Notch signalling to be initiated. AAJs, which form at a similar time to the onset of *m5/m8* transcription, appear as normal in *nullo* mutants, unlike BAJs, and could be crucial for Notch activation (Hunter and Wieschaus, 2000). Therefore, we next investigated the consequences on *m5/m8* directed transcription of disrupting all AJs, by depleting the key junctional linker α-Catenin (α-Cat) (Staller et al., 2013). Maternal RNAi knockdown (KD) led to a marked depletion of α*-Cat* mRNA and protein (Fig. S6A,B), resulting in 100% embryos with gastrulation failure but with only modest delays in cellularization (Fig. S6C). Strikingly, Notch-dependent transcription was affected in these α-Cat KD embryos in advance of any gastrulation defects. The main consequences were a disruption of the mesectodermal stripe (Fig. 6A) and an overall reduction in the mean levels of transcription without affecting the onset times (Fig. 6C,D, Movie 5). This was due to a shift in the distribution of activity levels, with many nuclei exhibiting a marked reduction in their overall mRNA output (Fig. 6E, Fig. S6D). To determine whether α-Cat contribution to Notch signalling is relevant in the context of endogenous gene activity, we tagged with MS2 loops one of the Notch target genes proposed to be regulated by the *m5/m8* enhancer – *E(spl)m8-HLH* (Zinzen et al., 2006). In a similar way to *m5/m8^[III]^*, *E(spl)m8-HLH* transcription was disrupted upon α-Cat KD: the mesectodermal stripe was disorganized, the mean levels were reduced without a change in onset times, and the range of accumulated mRNA levels per nucleus was diminished (Fig. 6B,F-H, Fig. S6E, Movie 6). Overall, these results suggest that the formation of AJs is an important step in the timing and strength of Notch activation during nc14. When perturbed, reduced levels of Notch-dependent transcription occurred.

**Fig. 7.**
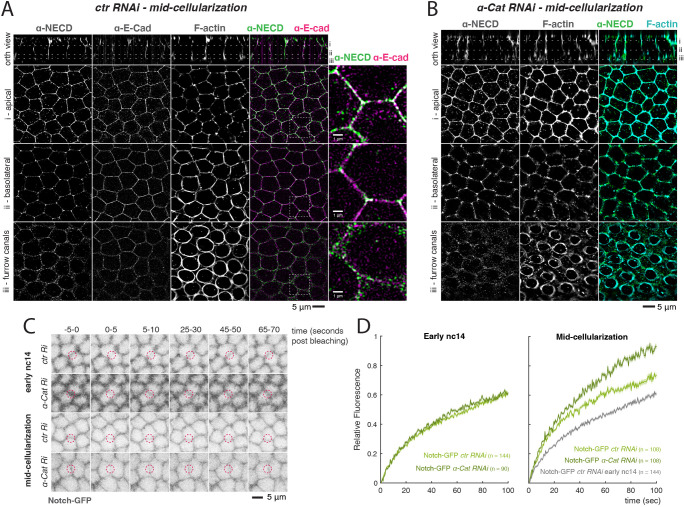
**α-Catenin depletion influences Notch membrane dynamics but not localization.** (A,B) Mid-cellularization control (A) or *α-Cat* (B) RNAi embryos stained with phalloidin and antibodies against the extracellular domain of Notch (NECD) and E-cad, and imaged using SIM (E-cad channel not shown in B). Top panels are orthogonal views with lines marking individual planes shown below. (C) Stills of Notch-GFP FRAP experiments in the indicated conditions in early nc14 or mid-cellularization embryos. Each still is an average of 10 frames between the indicated timepoints. Red circles indicate the quantified region over time. (D) FRAP experiments performed on Notch-GFP in early nc14 (left) and mid-cellularization (right) embryos comparing control with α-Cat depletion. Notch-GFP is expressed at endogenous levels from a genomic BAC (Couturier et al., 2012).

To investigate whether the role of α-Catenin and AJs was likely to involve direct effects on Notch, we used SIM (structured illumination microscopy) to assess the extent of protein colocalization. The high-resolution imaging revealed a heterogenous distribution of Notch along the growing lateral membranes. Apically, Notch levels were similar around the whole circumference, whereas sub-apically Notch was enriched at tricellular junctions and more basally it was present in the furrow canals (the most basal part of growing membranes), delineated by F-actin (Fig. 7A). E-cadherin was also detected in all these positions, but the two proteins were distributed unevenly in membrane clusters with relatively few sites where they were colocalized (Fig. 7A). Overall, the low level of colocalization suggests that Notch is not directly sequestered into the AJs, although it is in close proximity. Furthermore, Notch localization was not disrupted upon α-Catenin depletion. In embryos at mid-cellularization (around the time at which Notch-dependent transcription initiates), Notch was present at a similar level and with similar overall distribution in α-Catenin-depleted embryos (Fig. 7B, Fig. S7B). Although defects in adhesion became evident at late cellularization, in the form of ‘holes’ at the tricellular junctions (Yu and Zallen, 2020) that also displaced Notch into a surrounding ring (Fig. S7A), no other changes in Notch localization were apparent, leading us to conclude that α-Catenin depletion does not generally disrupt the distribution of Notch in the lateral membranes, despite its effect on Notch-dependent transcription. Similar results were obtained from live-imaging Notch-GFP and Dl- mScarlet in α-Catenin-depleted embryos where there was no change in the distribution of either protein (Fig. S7B).

α-Catenin has been proposed to influence E-cad stability at the membrane (Bajpai et al., 2008; Jurado et al., 2016; Ishiyama et al., 2018). We thus wondered whether α-Catenin depletion could similarly be influencing Notch stability, rather than localization. To this end, we measured the fluorescence recovery after photobleaching (FRAP) of Notch-GFP expressed at endogenous levels (Couturier et al., 2012), as an indication of its turnover in the membrane. We were unable to perform similar experiments with Dl-mScarlet because it bleached too rapidly. There was a notable change in the speed of recovery for Notch-GFP between early nc14 and mid-cellularization time points, with faster recoveries detected at the later time point, suggesting there is more rapid turnover of Notch in the membrane around the time that signalling commences (Fig. 7C,D). However, as the measurements were made at random locations in the embryo, the differences represent general properties of Notch at this time, rather than any signalling-induced changes, as the latter would be restricted to mesectodermal cells. α-Catenin depletion had no effect on the Notch recovery at the early time point. However, at mid-cellularization, α-Catenin depletion resulted in faster recovery times (Fig. 7C,D), suggesting that it normally restricts the turnover or recycling. One consequence would be that, in wild-type embryos, Notch would have a longer residence time in the membrane, which could permit higher levels of signalling to be attained.

## DISCUSSION

The geometry of a tissue and the nature of the cell contacts are likely to be important factors influencing the levels and duration of Notch signalling (Shaya et al., 2017). By analysing the transcriptional output of Notch signalling in live blastoderm embryos, we have been able to relate the time of productive ligand-receptor interactions with landmarks in cellular membrane growth. Strikingly, signalling was initiated after lateral membranes had grown to approximately one-third of their final length but before cellularization was complete. There was a strong correlation between cellularization time in each embryo, measured by the length of the lateral membranes, and onset of transcription, even in embryos where membrane growth was delayed. These results argue that a key step during membrane morphogenesis determines when signalling can initiate. The same restrictions could also influence when signalling can re-initiate following cell division.

The requirement for lateral membrane growth and morphogenesis can help to explain why two different Notch-responsive enhancers initiate transcription within a few minutes of each other (Falo-Sanjuan et al., 2019), because there would be a coordinated release of NICD when the receptor and ligands first became juxtaposed. It is also plausible that the lateral membranes are essential for the activity of Neuralized, an E3-ligase that is essential for Delta endocytosis and activation, the expression of which commences in the mesoderm cells at this time (Price et al., 1993). Furthermore, the correlations, together with the lack of Notch-dependent transcription in *slam* mutant embryos where lateral membranes are arrested, are hard to reconcile with the model that NICD accumulates in the nucleus from the beginning of nc14, as has been suggested (Viswanathan et al., 2019). Our results also favour the model that signalling is initiated *in trans*, between receptor and ligand located on neighbouring cell membranes, rather than *in cis*, between ligand and receptors on the same apical and/or internal membranes (Coumailleau et al., 2009; Nandagopal et al., 2019), a model that is also consistent with the requirement for Neuralized in the neighbouring mesoderm.

One plausible explanation for the precise onset of transcription at a specific moment during membrane morphogenesis could be that a minimal area of interface is required for signalling to surpass a crucial threshold. However, our data argue against the membrane area being the limiting factor and suggest that the formation and/or maturation of membrane domains or junctions is required. First, the transcription onsets and lateral membrane growth were no longer strongly correlated in *nullo* mutants. Second, Notch-responsive transcription was impaired when α-Cat, a key component of AJs, was depleted. The number of nuclei with high levels of transcription from the *m5/m8* enhancer was reduced in these embryos, leading to a reduction in the overall mean levels. Similar effects on the endogenous *E(spl)m8-HLH* were also seen upon α-Cat depletion. As the lateral membranes are fully formed in the α-Cat-depleted embryos, the results suggest that features coordinated by AJs are important for normal signalling. Given the variability of the effects on transcription, it is likely that these properties are required to achieve high levels of Notch signalling, rather than being absolutely required for Notch activation.

The effects of AJs on Notch signalling could be direct or indirect. Based on super-resolution imaging, there was no specific co-enrichment of Notch with components of AJs, such as Cadherin, nor was Notch localization adversely affected by α-Cat depletion. Together, these results make it unlikely that the direct recruitment of Notch to apical junctions is a limiting factor. However, Notch dynamics at the membrane were altered in α-Cat-depleted embryos, based on FRAP experiments. These indicated that the membrane-associated Notch is less stable when α-Cat is depleted, which could reduce the amount of Notch that is available to interact and signal at any one moment (Khait et al., 2016). It is not possible to distinguish whether the altered dynamics are due to changes in recycling and/or synthesis or in lateral diffusion. As the last could also result in altered segregation of Notch and the γ-secretase cleavage machinery (Kwak et al., 2020 preprint), all changes could explain the reduced transcription output in the α-Cat-depleted embryos. Alternative explanations are that α-Cat, and AJs, contribute to Notch activation because they bring the neighbouring membranes into sufficient proximity or because of their role in cell-cell adhesion. α-Cat functions as the linker between AJs and actomyosin, and is involved in transmitting contractile forces across cells (Jurado et al., 2016). AJ-mediated adhesion could promote higher Dl-pulling force, thus enhancing Notch cleavage and NICD release (Gordon et al., 2015) to regulate outputs. It is also possible that α-Cat exerts its effects via a combination of mechanisms.

Our data that lateral membranes are required for signalling are consistent with elegant experiments tracking photoconverted receptor populations in *Drosophila* sensory organ precursors (SOPs), which indicated that the lateral pool of Notch is the one that becomes activated (Trylinski et al., 2017). In this context, the active receptor population was located basal to the apical junctions. In contrast, during vertebrate neurogenesis, adherens junctions at the apical luminal surface of the neuronal progenitors have been proposed as the site of signalling (Hatakeyama et al., 2014). As Notch does not strongly colocalize with Cadherin at cellularization, our results fit better with those from SOPs and from cell culture studies proposing that full-length Notch is excluded from AJs (Kwak et al., 2020 preprint). However, ligand interactions and post-activation cleavage may occur at different sites in the membrane and indeed the sites of ligand interactions may differ according to the tissue architecture. For example, in the *Drosophila* follicular epithelium, cells receive signals from the neighbouring germ cells via their apical surface (Lopez-Schier and St Johnston, 2001). In other contexts, basal actin-based protrusions and cytonemes have been proposed as the ligand source that mediates longer range signalling (Huang and Kornberg, 2015; Hunter et al., 2019; Boukhatmi et al., 2020). Nevertheless, it is evident from the results presented here that the cell architecture, and the formation of apical junctions, are important features in enabling signalling in a simple epithelium. It will be interesting to see in which other contexts adherens junctions contribute to Notch activity. For example, a recent study showed AJ disruption in the mouse brain led to a phenotype of early differentiation of progenitor cells similar to that caused by reduced Notch signalling (Kwak et al., 2020 preprint), suggesting there might be a widespread role of AJs in modulating Notch activity.

## MATERIALS AND METHODS

### Fly strains and genetics

The following *Drosophila* strains were used: *sqh-Gap43-mCherry* (Izquierdo et al., 2018), *GFP-gish[Spider]* (BDSC #59025, Morin et al., 2001), *shg-GFP* (BDSC #60584, Huang et al., 2009), *Notch-GFP* (*Ni-GFP* from Couturier et al., 2012), *Dl-mScarlet* (Boukhatmi et al., 2020), *Nup107-GFP* (BDSC #35514, Katsani et al., 2008), *nos-MCP-GFP* (II, BDSC #63821) and *His2Av-RFP*; *nos-MCP-GFP* (BDSC #60340, Garcia et al., 2013), and *His2Av-RFP* (III, BDSC #23650). The *m5/m8-peve-MS2-lacZ* second chromosome (*m5/m8^[II]^*) and third chromosome (*m5/m8^[III]^*) MS2 reporter lines were generated by Falo-Sanjuan et al. (2019). *E(spl)m8-HLH- MS2* was generated during this work. Full genotypes of used lines are detailed in Table S1.

### Generation of endogenously tagged E(spl)m8-HLH-MS2

Twenty-four MS2 loops, lacZ and SV40 (5.4 kb in total, same as used for the *m5/m8* reporter) were inserted in the genome by CRISPR/Cas9 scarless genome engineering (flycrispr.org) to replace the *E(spl)m8-HLH-MS2* 3′UTR, while keeping its coding sequence intact. Briefly, a plasmid containing homology arms flanking *E(spl)m8-HLH-MS2* 3′UTR, lacZ, SV40 and the PiggyBac 3xPax3-dsRED cassette from *pHD-ScarlessDsRed* (flycrispr.org) was synthesized by NBS Biologicals. Twenty-four MS2 loops from *pCR4- 24XMS2SL-stable* (Addgene #31865) were subsequently inserted using an *EcoR*I site. Transformants were obtained by co-injecting (performed by the Genetics Fly Facility, University of Cambridge, UK) this plasmid with a pCFD3-dU6:3gRNA plasmid (Addgene #49410) expressing the gRNA *CTGTGATAGCCCAACTGTGA* and screening for 3xPax3-dsRED. The *3xPax3-dsRED* cassette was excised by crossing with αTub84B-PiggyBac flies (BDSC #32070). Maps of the homology and gRNA plasmids and final genomic sequence can be found at https://benchling.com/braylab/f/tE0Fz0Q1-endogenous-ms2-lines/.

### Mutant backgrounds

To test expression from *m5/m8* in the *kuk[PE]* mutant background, a second chromosome recombinant *His2av-RFP, nos-MCP-GFP* (Falo-Sanjuan et al., 2019) was combined with *kuk[EY07696]* (BDSC #16856, Pilot et al., 2006). *m5/m8^[II]^* was also combined with *kuk[EY07696]* and, as *kuk[EY07696]* is homozygous viable, *His2av-RFP, nos-MCP-GFP* / CyO ; *kuk[EY07696]* females were crossed with *m5/m8^[II]^*; *kuk[EY07696]* males to obtain embryos that were maternal and zygotic mutant for this hypomorphic *kuk* allele. Control embryos were obtained by crossing *His2av-RFP, nos-MCP-GFP* / CyO females with *m5/m8^[II]^* males.

To test expression from *m5/m8* in the *slam* and *nullo* mutant backgrounds, third chromosome recombinants *His2av-RFP, nos-MCP-GFP* (Falo-Sanjuan et al., 2019) were combined with deficiencies encompassing *nullo* (*Df(1)Sxl-bt*, BDSC #3196) or *slam* (*Df(2L)Exel6016, Pw[+mC]=XP-UExel6016t*, BDSC #7502). *m5/m8^[III]^* was also combined with *Df[slam]*. Control embryos were obtained by crossing *His2av-RFP, nos-MCP-GFP* females with *m5/m8^[III]^* males. Homozygous mutant embryos for *slam* were obtained from crossing *Df[slam]* / *CTG*; *His2av-RFP, nos-MCP-GFP* with *Df[slam]* / *CTG*; *m5/m8^[III]^* and were recognized by the absence *CTG* (*CyO-twi-GFP*, BDSC #6662). Hemizygous embryos for *nullo* were obtained from crossing *Df[nullo]* / *FM6*;; *His2av-RFP, nos-MCP-GFP* with *m5/m8^[III]^* and were recognized by defects in gastrulation and lethality. All the mutant crosses yield one-quarter homozygous mutant progeny. In the remaining progeny, which were analysed in parallel, two-thirds would be heterozygous for each gene tested and one-third would not carry a mutation for the gene tested.

### Maternal KD

The maternal driver *αTub-Gal4::VP16* (BDSC # 7062) was combined with *His2av- RFP, nos-MCP-GFP* to generate *αTub-Gal4::VP16* ; *His2Av-RFP*, *nos-MCP-GFP*. To knock down α*-Cat* from the maternal germline, this stock was crossed with *UASp-α-Cat-RNAi* (BDSC #33430) or *UASp-w-RNAi* as control (BDSC #35573), and *αTub-Gal4::VP16*/+; *His2Av-RFP*, *nos-MCP-GFP*/*UASp-RNAi* females were crossed with *m5/m8^[III]^* to obtain the experimental embryos. To quantify the degree of maternal KD, *αTub-Gal4::VP16* was crossed with the same lines and F2 embryos were collected for antibody staining and RT-qPCR. Crosses used for each experiment are detailed in Table S2.

### Imaging

Embryos were collected on apple juice agar plates with yeast paste, dechorionated in bleach and mounted in Voltalef medium (Samaro) between a semi-permeable membrane and a coverslip. The ventral side of the embryo was facing the coverslip. Imaging was performed at 20-22°C.

Movies were acquired in a Leica SP8 confocal using a 40×apochromatic 1.3 objective, ×2 zoom and 400×400 pixel^2^ (providing an *xy* resolution of 0.36 μm/pixels), 12 bit depth, 400 Hz image acquisition frequency and pinhole of 4 airy units. In experiments where cellularization was quantified using the transmitted light channel, 33×2 mm slices were collected to reach the cross-section of the embryo, providing a time resolution of 20 s per frame. In other experiments, 29×1 mm slices were collected, with total acquisition time of 15-60 s per frame, depending on the experiment. Nup107-GFP movies were acquired using 4× zoom (0.18 μm/pixel in *xy*, 1 μm slices).

### Antibody staining

Embryos where dechorionated in bleach and fixed in a 1:1 mixture of heptane and 40% formaldehyde for 9 min. Embryos were then stuck to tape, manually devetillinized in PBS and transferred to eppendorf tubes. Staining to quantify maternal KD and for SIM were carried out in the same way: embryos were blocked in 1% BSA for 1 h, incubated with primary antibodies overnight at 4C, washed in PBS-Triton-X 0.1%, incubated with secondary antibodies for 2 h at room temperature, washed in PBS-Triton-X and mounted in Vectashield mounting medium. Primary antibodies were: 1:100 rat anti-DCAT-1 [Developmental Studies Hybridoma Bank (DHSB)], 1:10 mouse anti-NECD (C458-2H, DSHB) and 1:10 rat anti-DCAD2 (DCAD2, DSHB). Secondary antibodies were: 1:200 anti-Rat-FITC (712-095-153, Jackson ImmunoResearch) for α-Cat KD quantification; 1:200 anti-Mouse-Alexa488 (AB_2338189, Invitrogen) and 1:200 anti-Rat-Alexa568 (A-11077, ThermoFisher) for SIM. Embryos were also stained with 1:1500 Phalloidin-iFluor647 (ab176759, Abcam).

### mRNA extraction and qPCR

Embryos were dechorionated in bleach and early embryos (pre-nc10)/eggs were selected in Voltalef medium. Pools of 15-20 embryos of each genotype were transferred to eppendorf tubes and dissociated in TRI Reagent (Sigma) with a plastic pestle. mRNA was extracted by adding chloroform, followed by 10 min centrifugation at 4°C and precipitation with isopropanol overnight. DNA was then pelleted by 10 min centrifugation at 4°C, washed in 70% ethanol, dried and resuspended in DEPC-treated water. Approximately 2 mg of RNA from each sample were DNAse treated with the DNA-free DNA Removal Kit (Invitrogen) in the presence of RiboLock RNase Inhibitor (Thermo Scientific). 1 mg of DNA-free RNA was then used for reverse transcription using M-MLV Reverse Transcriptase (Promega) in the presence of RiboLock. Samples were diluted 1:2 for RT-qPCR using SYBR Green Mastermix (Sigma) and primers detailed in Table S3.

### Structured illumination microscopy

Structured Illumination Microscopy (SIM) was carried out in stained samples prepared as detailed above, in a Zeiss Elyra 7 Lattice SIM microscope, using a 63×1.4 NA immersion oil objective. Three-colour Lattice SIM stacks were acquired with a 110 nm step size and reconstructed using the ZEN software (Zeiss). The final *xy* resolution of super-resolved images was 31.3×31.2 nm/pixel (2560×2560 pixel^2^).

### FRAP

Imaging of Notch-GFP was performed as for live imaging, but using a 4× zoom (0.18 mm/pixel *xy* resolution), 400×400 pixel^2^ size. Point bleaching was performed at six points that targeted membranes per round of FRAP for 0.5 s each (total bleach time 3 s) simultaneously with 488 and 561 nm laser. Pre and postbleaching images were collected at 400 Hz (0.5 s/frame). FRAP was quantified by drawing circles of 20 pixels in diameter around the bleached regions and at another six control regions in non-bleached membranes. FRAP recovery was calculated by dividing the average fluorescence at each region by the average pre-bleach intensity and normalized for the ratio of the average fluorescence at control regions to pre-bleach intensity, to account for loss of fluorescence due to bleaching during acquisition (Gomez-Lamarca et al., 2018). Each curve was then scaled so that the first value after beaching was considered 0. Attempts at FRAP with Dl-mScarlet were unsuccessful because it bleached very rapidly and recovery could not be accurately quantified.

### Image analysis

#### Quantifying membrane length

Length of membranes during cellularization was calculated from the orthogonal section in the centre of the field of view. Fluorescent signal was thresholded using the Otsu method (Otsu, 1979) and the height of the obtained object, equivalent to the length of the membrane at each time point, was calculated and plotted over time. Because the signal:noise ratio was different for each marker used, these quantifications were manually curated by marking the extent of membrane signal in the orthogonal view image when the automated segmentation did not match the raw signal.

#### Tracking nuclei and MS2 quantification

Movies were analysed using custom MATLAB (MATLAB R2020a, MathWorks) scripts that have been previously described (Falo-Sanjuan et al., 2019), with some adaptations. Briefly, the His2Av-RFP signal was used to segment and track the nuclei in 3D. Each 3D stack was first filtered using a 3D median filter of three and increasing the contrast based on the intensity profile of each frame to account for bleaching. A Fourier transform log filter was then applied to enhance round objects (Garcia et al., 2013). Segmentation was performed by applying a fixed intensity threshold to the filtered stack, which was empirically determined. This was followed by filters to fill holes in objects and discard mis-segmented nuclei based on size. 3D watershed accounting for anisotropic voxel sizes (Mishchenko, 2015) was used to split merged nuclei. The final segmented stack was obtained by filtering by size again and thickening each object. Last, the segmented stack was labelled to assign a number to each object, and the position of each centroid (in *x*, *y* and *z*) was calculated for tracking.

Nuclei were then tracked in 3D by finding the nearest object (minimum Euclidean distance between two centroids in space) in the previous two frames, which was closer than 6 μm. If no object was found, that nucleus was kept with a new label. If more than one object was ‘matched’ with the same one in one of the previous two frames, both were kept with new labels.

After tracking, the positions of all pixels from each nucleus in each frame were used to measure the maximum fluorescence value in the GFP channel, which was used as a proxy of the spot fluorescence. When a spot cannot be detected by eye, this method detects only background, but the signal:background ratio is high enough that the subsequent analysis allows confident classification of when the maximum value is really representing a spot.

#### Nuclear membrane tracking

To segment the nuclei in 3D from nuclear membrane markers (Nup107-GFP), each 3D stack was first resized to produce 1:1:1 ratio voxel sizes using the cubic interpolation from the *imresize3* function in MATLAB. Each resized stack was then filtered using a 3D gaussian filter of 1. To account for loss of fluorescence due to bleaching, the *imhistmatchn* function was used to adjust the contrast of each frame to the first one. A fixed intensity threshold of 10% was used to create a thresholded image, which was used as seed for Active Contour segmentation (Chan and Vese, 2001) of the filtered image to produce an initial segmentation of nuclear membranes. The image was then inverted to recognize as object the space inside the nuclear membrane rather than the membrane itself. A filter based on the proportion of object present in each slice was used to remove the vitelline membrane. A 3D watershed filter was then used to separate merged objects, and object thickening was used to compensate for any signal that was lost at the edges. Finally, 3D objects out of the range 10 mm^3^ to 200 mm^3^ were discarded. Segmented nuclei were then tracked in 3D, as described in the previous section. In this case, because more nuclei were missing in each frame than when histones were segmented, a maximum distance of 4 mm was allowed for a nucleus to be considered the same as another in a maximum of five previous frames.

#### Nuclear 3D properties

After tracking, the MATLAB function *regionprops3* was used to extract 3D properties of each object: volume, surface area, solidity and length of principal axes. 2D slices at different fractions of the nuclear length (25, 50 and 75%) were extracted and 2D properties quantified using *regionprops*: area, perimeter and eccentricity. The slices were calculated on a per embryo basis, which means that they will not correspond to precisely the same position in all nuclei, owing to the curvature. As almost all nuclei were imaged in the same plane; the 2D properties measured will not have been substantially affected by this generalization. The same approach to measure size and shape of nuclei was employed with His2Av labelling. This provided a good approximation of the volume and length of the nuclei but the fine details of the nuclear wrinkling could not be resolved.

### Data processing and statistical analysis

#### MS2 data processing

Processing of MS2 data (definition of active nuclei and normalization for bleaching) has been carried out as described in our previous work (Falo-Sanjuan et al., 2019). From the tracking step, the fluorescent trace of each nucleus over time was obtained. Only nuclei tracked for more than 10 frames were retained. First, nuclei were classified as inactive or active. To do so, the average of all nuclei (active and inactive) was calculated over time and fitted to a straight line. A median filter of three was applied to each nucleus over time to smooth the trace and ON periods were considered when fluorescent values were 1.2 times the baseline at each time point. This produced an initial segregation of active (nuclei ON for at least five frames) and inactive nuclei. These parameters were determined empirically on the basis that the filters retained nuclei with spots close to background levels and excluded false positives from bright background pixels. The mean fluorescence from MCP-GFP in the inactive nuclei was then used to define the background baseline and active nuclei were segregated again in the same manner. The final fluorescence values in the active nuclei were calculated by removing the fitted baseline from the maximum intensity value for each, and normalizing for the percentage that the MCP-GFP fluorescence in inactive nuclei decreased over time to account for the loss of fluorescence due to bleaching. Nuclei active in cycles before nc14 were discarded based on the timing of their activation.

In all movies, time into nc14 was considered from the end of the 13th syncytial division. Onsets of transcription were defined as the beginning of the first ON period, starting from 15 min into nc14 in most experiments, except for expression in the presence of maternal Gal4 (expression from 30 min to exclude earlier stochastic activity). The total mRNA output (in AU) was obtained by adding all the normalized transcription values for each cell in a defined time period. Cells producing ‘high’ and ‘low’ total mRNA output were defined by values that were above and below the median.

#### Statistical analysis

In figures and figure legends, *n* indicates number of embryos imaged for each biological condition. Where appropriate, *n* next to heatmaps indicates total number of cells combining all embryos for each biological condition. Plots showing mean levels of transcription and s.e.m. combine all traces from multiple embryos from the same biological condition.

### Reagents and software availability

Modifications in the existing code to track nuclei from the nuclear membrane signal and quantify nuclear morphology in 3D and 2D slices have been incorporated in a MATLAB app and can be obtained from https://github.com/BrayLab/LiveTrx.

## Supplementary Material

Supplementary information

Reviewer comments
