# Supplementary material for: Membrane architecture and adherens junctions contribute to strong Notch pathway activation
Source: Development. 2021 Oct 14;148(19):dev199831. doi: 10.1242/dev.199831 (PMC8543148; doi:10.1242/dev.199831)
Supplement: Supplementary information [file develop-148-199831-s1.pdf]

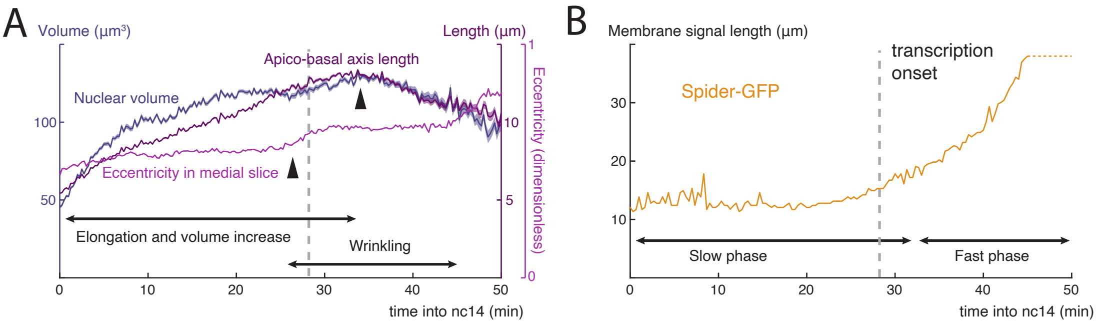

Fig. S1. Correlation between developmental processes and onset of Notch dependent transcription. A) Timeline of changes in nuclear 3D properties over time, quantified using the nuclear membrane marker Nup107-GFP. **B**) Timeline of cellularization, measured by quantification of the length of the cell membrane marker Spider-GFP in orthogonal views. Dashed lines indicate length of signal is greater than the stack imaged.

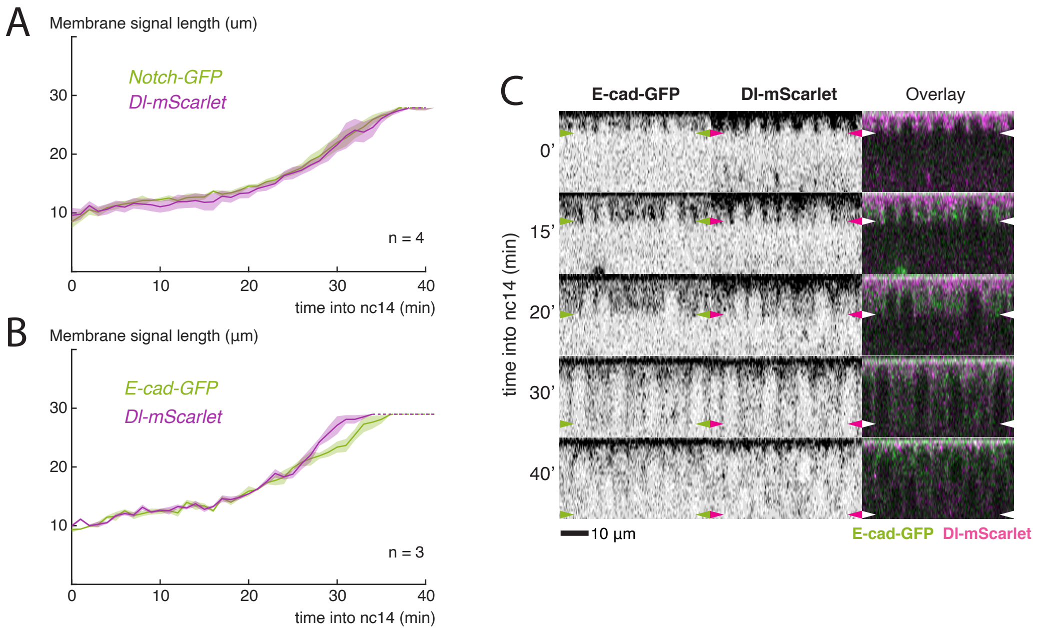

Fig. S2. Delta tracks with E-cadherin as membranes grow. A) Comparison of the length of membrane occupied by Notch and Delta over time, extending basally at the same rate. **B**) Comparison of the length of lateral signal of E-cad and Delta over time. Dashed lines indicate membrane length is greater than the stack imaged. **C**) Orthogonal views from embryos expressing E-cad-GFP and Dl-mScarlet, showing colocalization at all timepoints during cellularization. Arrowheads indicate position of cellularization front.

Development • Supplementary information

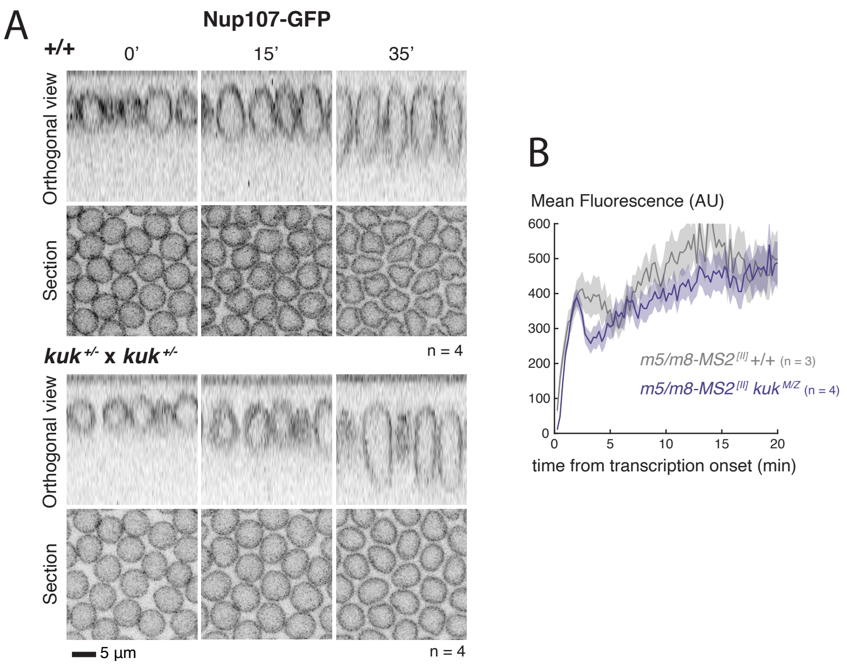

Fig. S3. Changes in nuclear morphology do not influence Notch dependent transcription. A) Cross-sections and orthogonal views of the nuclear membrane marker Nup107-GFP in wild type (top) and embryos obtained from *kuk* heterozygous parents (bottom) at the indicated times (min into nc14), as this *kuk* allele was not homozygous viable in combination with Nup107-GFP. **B**) Mean levels of transcription when nuclei are aligned by onset times. Mean and SEM (shaded area) of all cells combined from multiple embryos are shown (n embryo numbers indicated in each).

Development • Supplementary information

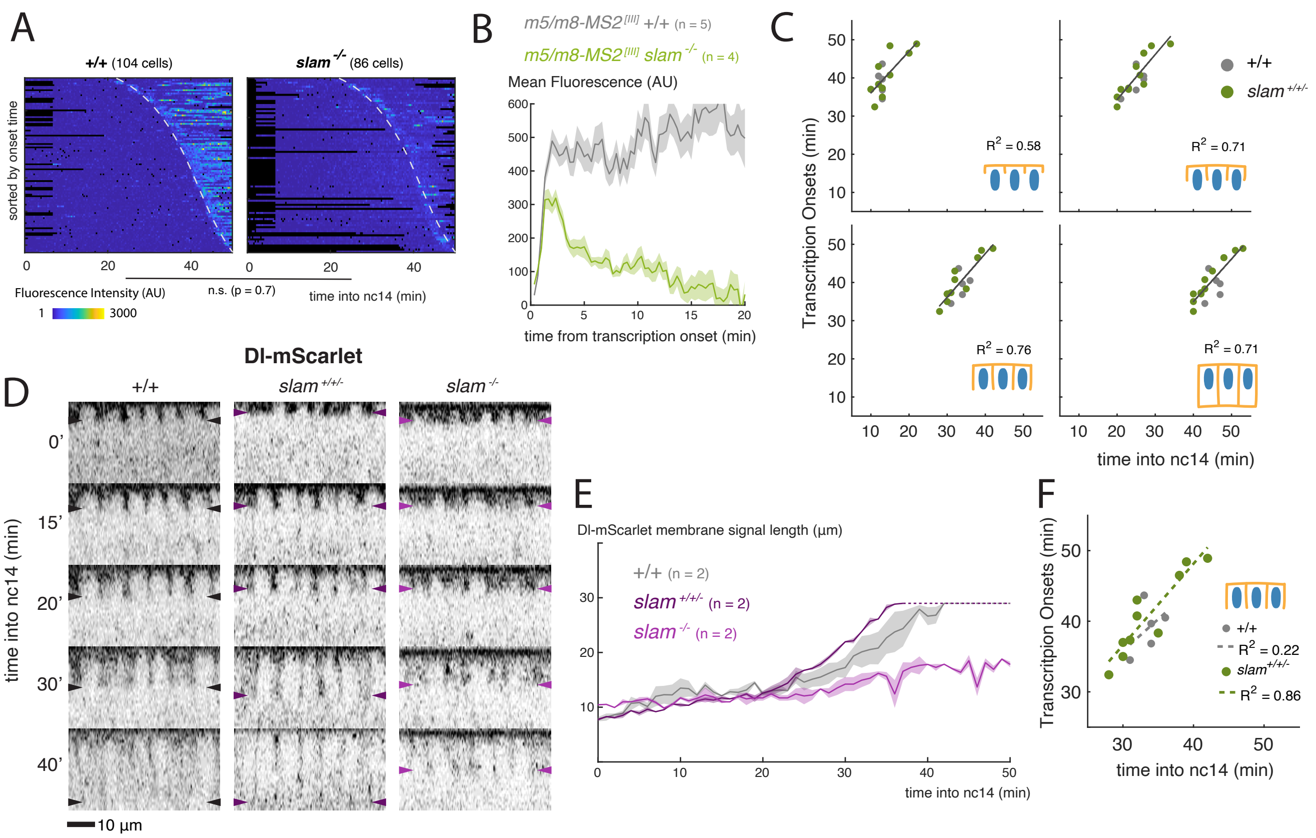

Fig. S4. Delta localization in *slam* mutant embryos. A) Heatmaps of transcription in all mesectoderm nuclei from control and *slam*$^{-/-}$ embryos, sorted by onset time. Dashed lines in-dicate onset times in controls. n.s.: not significant, p-value calculated using Kolmogorov–Smirnov test. **B**) Mean levels of transcription in *slam*$^{-/-}$ embryos compared to controls when nuclei are aligned by their onset times. **C**) Correlation between timepoints during cellularization (indicated by each cartoon) with onset of transcription from *m5/m8* [III] (calculated as the first quartile of onset times) in *slam*$^{+/+/-}$ and control embryos. R^2 coefficients are calculated after pooling all points shown the same plot together. **D**) Orthogonal views from embryos expressing Dl-mScarlet in wild type, *slam*$^{-/-}$ or *slam*$^{+/+/-}$ backgrounds. Arrowheads indicate position of the most basal signal. **E**) Comparison of the length of membrane localization of Dl-mScarlet in wild type, *slam* homozygous embryos and other embryos obtained from the same cross (*slam*$^{+/+/-}$). Delta did not extend basally in *slam*$^{-/-}$ embryos. In **B** and **E** mean and SEM (shaded area) are shown, n embryos indicated for each. Dashed lines indicate membrane length is greater than the stack imaged. **F**) Correlation between the timepoint of cellularization when membranes reach the basal end of nuclei with onset of transcription from *m5/m8* [III] (calculated as the first quartile of onset times) in *slam*$^{+/+/-}$ and control embryos. R^2 coefficients are calculated independently for each genotype, indicated by dashed lines.

Development • Supplementary information

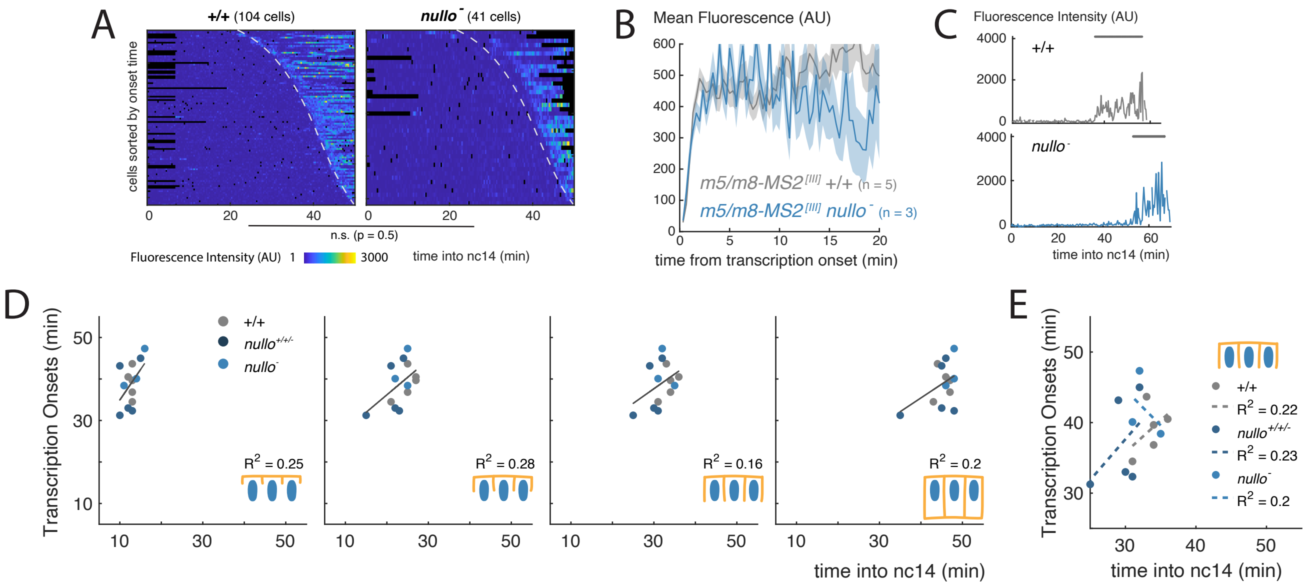

Fig. S5. Absence of Nullo does not affect overall levels of transcription. **A**) Heatmaps of *m5/m8* [*III*] transcription in all mesectoderm nuclei sorted by onset time. Dashed lines indicate onset times in wild type. n.s.: not significant, p-value calculated using Kol-mogorov–Smirnov test. **B**) Mean levels of transcription when nuclei are aligned by their onset times. **C**) Examples of transcription traces from mesectoderm nuclei. Grey lines indicate ON periods. **D**) Correlation between timepoints during cellularization (indicated by each cartoon) with onset of transcription from *m5/m8* [*III*] (calculated as the first quartile of onset times) in *nullo*$^{+/+/-}$, *nullo*- and control embryos. R^2 coefficients are calculated after pooling all points shown the same plot together. In **B**, mean and SEM (shaded area) of all cells combined from multiple embryos are shown (n embryo numbers indicated in each). **E**) Correlation between the timepoint of cellularization when membranes reach the basal end of nuclei with onset of transcription from *m5/m8* (calculated as the first quartile of onset times) in *nullo*$^{+/+/-}$, *nullo*-and control embryos. R^2 coefficients are calculated independently for each genotype, indicated by dashed lines. Images, plots and quantifications of control embryos are duplicated from **Fig.** 4.

Development • Supplementary information

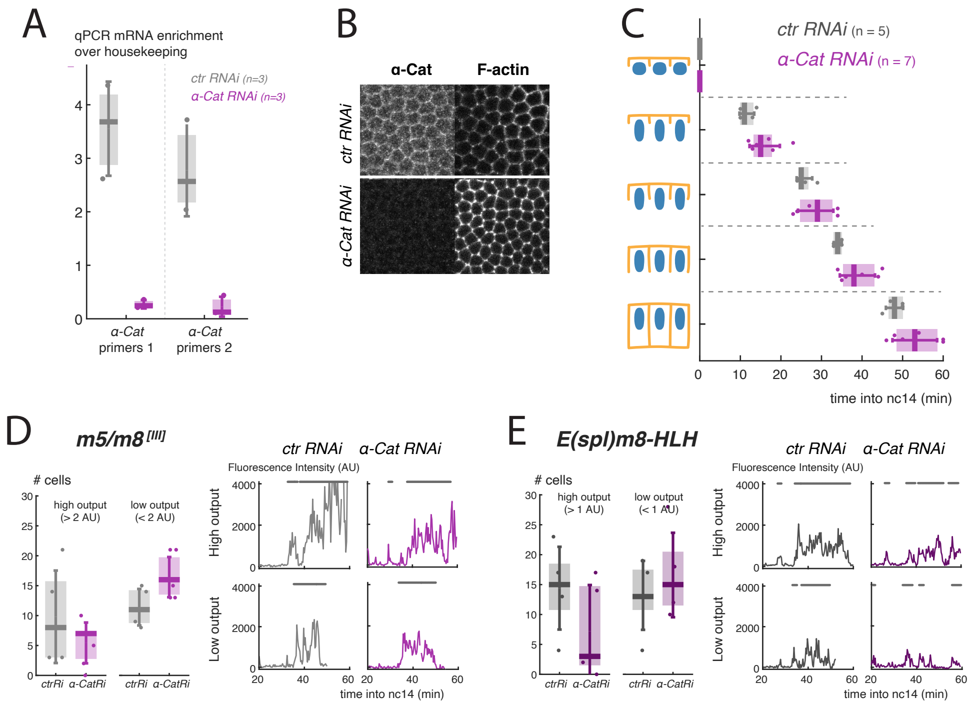

Fig. S6. Adherens junctions influence Notch dependent transcription. A) Quan-tification of *α-Cat* mRNA levels by RT-qPCR (2 sets of primers) in pools of 15-20 eggs and/or pre-nc13 embryos upon control and *α-Cat* germline RNAi expression. n = 3 (control RNAi) and 3 (*α-Cat RNAi*) biological replicates. **B**) Mid-cellularization embryos stained for α-Cat and F-actin (phalloidin) upon control and *α-Cat* germline RNAi expression. **C**) Boxplots indicating timing of cellularization progression (timepoints when membranes reach each of the lengths with respect to nuclei indicated in the cartoons) in control and *α-Cat RNAi* conditions, quantified from *m5/m8* [III] MS2 movies. Median, Q1/Q3 quartiles and SD shown. **D-E**) Boxplots indicating number of cells producing high and low total levels of transcription (left, defined by production above and below the median) and examples of transcription traces from each group (right), for *m5/m8* [III] (**D**) and *E(spl)m8-HLH* (**E**). Grey lines indicate ON periods.

Development • Supplementary information

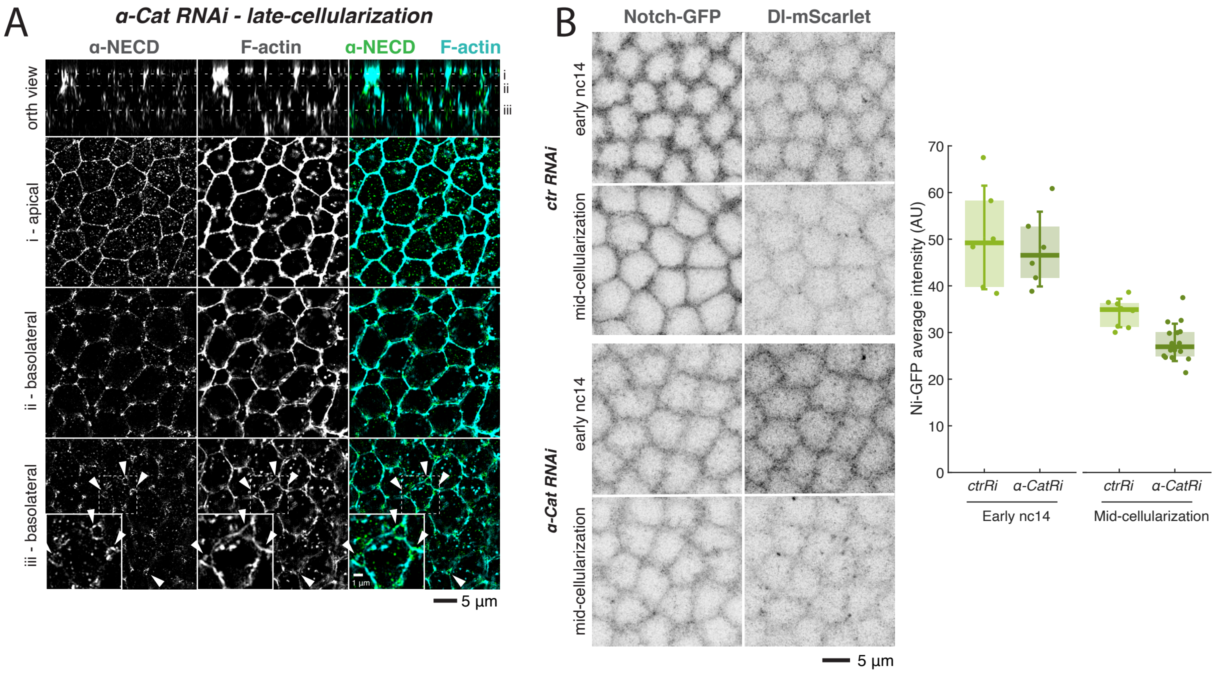

Fig. S7. α-Catenin depletion does not influence Notch localization. A) Late-cellularization *α-Cat* RNAi embryo stained with phalloidin and antibodies against NECD and E-cad and imaged using SIM (E-cad channel not shown). Arrowheads indicate holes in tricellular junctions caused by lack of adhesion. Top panels are orthogonal views with lines marking individual planes shown below. **B**) Stills of live early nc14 and mid-cellularization embryos expressing Notch-GFP and Dl-mScarlet upon control and *α-Cat* RNAi expression (left) and quantification of the overall Notch-GFP levels in each condition and timepoint (right). n = 6 (control RNAi early), 6 (*α-Cat* RNAi early), 8 (control RNAi mid-cellualarization) and 16 (*α-Cat* RNAi mid-cellularization).

Table S1. Full genotypes of used *Drosophila* lines

Name (Chr)	Full genotype	Source
His2Av::RFP (III)	w[*];P{w[+mC]=His2Av-mRFP1}III.1	BDSC #23650
His2Av::RFP; nos-MCP::GFP	y[1] w[*]; P{w[+mC]=His2Av-mRFP1}II.2; P{w[+mC]=nos-MCP.EGFP}2	BDSC #60340
nos-MCP::GFP (II)	y[1] w[*]; P{w[+mC]=nos-MCP.EGFP}8	BDSC #63821
αTub-Gal4::VP16 (II)	w[*]; P{w[+mC]=matalpha4-GAL-VP16}V2H	BDSC #7062
m5/m8-MS2 (II)	w; P{w[+mC]=m5/m8-peve-24xMS2-lacZ-SV40}attP40	*(Falo-Sanjuan et al. 2019)*
m5/m8-MS2 (III)	w; P{w[+mC]=m5/m8-peve-24xMS2-lacZ-SV40}attP2	*(Falo-Sanjuan et al. 2019)*
E(spl)m8-HLH-MS2 (III)	w; {24xMS2-lacZ-SV40}E(spl)m8-HLH-3'UTR	This work
Gap43::mCherry (I)	Pw[+mC]=sqhp-Gap43::mCherry	*(Izquierdo et al. 2018)*
Nup107::GFP (II)	w[*]; P{w[+mC]=GFP-Nup107.K}9.1	BDSC #35514
DEcad::GFP (II)	y[1] w[*]; TI{TI}shg[GFP]	BDSC #60584
spider::GFP (III)	w[*]; Pw[+mC]=PTT-GBgish[Spider]	BDSC #59025
Ni::GFP (II)	N[55e11] w[1118] / Y ; M[3xP3-RFP.attP.w+.NiGFP]51D / +	*(Couturier et al. 2012)*
Dl::mScarlet-I (III)	TI{TI}DlmScarlet-I	*(Boukhatmi et al. 2020)*
Df[slam] (II)	w[1118]; Df(2L)Exel6016, P{w[+mC]=XP-U}Exel6016/CyO	BDSC #7502
Df[nullo] (I)	wDf(1)Sxl-bt, y[1]/Binsinscy	BDSC #3196
kuk[PE] (III)	y[1] w[67c23]; P{w[+mC] y[+mDint2]=EPgy2}kuk[EY07696]	BDSC #16856
CTG	w[1118]; In(2LR)Gla, wg[Gla-1]/CyO, Pw[+mC]=GAL4-twi.G2.2, Pw[+mC]=UAS-2xEGFPAH2.2	BDSC #6662
w RNAi Valium22 (III)	y[1] sc[*] v[1]; P{y[+t7.7] v[+t1.8]=TRiP.GL00094}attP2	BDSC #35573
α-Cat RNAi Valium20 (III)	y[1] sc[*] v[1] sev[21]; P{y[+t7.7] v[+t1.8]=TRiP.HMS00317}attP2	BDSC #33430

Development • Supplementary information

Table S2. Genotypes used in each experiment

Cross	Figure
♀ *His2Av::RFP* ; *nos-MCP::GFP* x ♂ *m5/m8-peve-MS2-lacZ-SV40*[attP40,II]	1**B**
♀ x ♂ *Nup107::GFP*	1**C**, S1**A**, S3**A**
♀ x ♂ *Spider::GFP* ; *His2Av-::RFP*	1**B**
♀ x ♂ *Ni::GFP* / + ; *Dl::mScarlet* / +	2**A**, S2**A**
♀ *Gap43::mCherry* ;; *nos-MCP::GFP* x ♂ *m5/m8-peve-MS2-lacZ-SV40*[attP40,II]	2**B**
♀ x ♂ *nos-MCP::GFP* / *m5/m8-peve-MS2-lacZ-SV40*[attP40,II] ; *Dl::mScarlet* / +	2**C**
♀ x ♂ *ECad::GFP* / + ; *Dl::mScarlet* / +	S2**B**, **C**
♀ *His2Av::RFP*, *nos-MCP::GFP* / CyO x ♂ *m5/m8-peve-MS2-lacZ-SV40*[attP40,II]	3, S3**B**
♀ *His2Av::RFP*, *nos-MCP::GFP* / CyO ; *kuk[PE]*<br>x ♂ *m5/m8-peve-MS2-lacZ-SV40*[attP40,II] ; *kuk[PE]*	3, S3**B**
♀ x ♂ *Nup107::GFP* ; *kuk[PE]* / TM6B	S3**A**
♀ *His2Av::RFP*, *nos-MCP::GFP* (III) x ♂ *m5/m8-peve-MS2-lacZ-SV40*[attP2,III]	4, S4**AC**, 5, S5
♀ *Df[slam]* / CTG ; *His2Av::RFP*, *nos-MCP::GFP*<br>x ♂ *Df[slam]* / CTG ; *m5/m8-peve-MS2-lacZ-SV40*[attP2,III]	4, S4**AC**
♀ x ♂ *Df[slam]* / CTG ; *Dl-mScarlet* / TM6B	S4**DE**
♀ *Df[nullo]* / FM6 ;; *His2Av::RFP*, *nos-MCP::GFP*<br>x ♂ *m5/m8-peve-MS2-lacZ-SV40*[attP2,III]	5, S5
♀ *αTub-VP16* / + ; *His2Av::RFP*, *nos-MCP::GFP* / *UASp-w RNAi*<br>x ♂ *m5/m8-peve-MS2-lacZ-SV40*[attP2,III]	6**ACDE**, S6**CD**
♀ *αTub-VP16* / + ; *His2Av::RFP*, *nos-MCP::GFP* / *UASp-α-Cat RNAi*<br>x ♂ *m5/m8-peve-MS2-lacZ-SV40*[attP2,III]	6**ACDE**, S6**CD**
♀ *αTub-VP16* / + ; *His2Av::RFP*, *nos-MCP::GFP* / *UASp-w RNAi*<br>x ♂ *MS2-lacZ-SV40*[E(spl)m8-HLH-3'UTR]	6**BFGH**, S6**E**
♀ *αTub-VP16* / + ; *His2Av::RFP*, *nos-MCP::GFP* / *UASp-α-Cat RNAi*<br>x ♂ *MS2-lacZ-SV40*[E(spl)m8-HLH-3'UTR]	6**BFGH**, S6**E**
♀ x ♂ *αTub-VP16* / + ; *UASp-w RNAi* / +	S6**AB**, 7**A**
♀ x ♂ *αTub-VP16* / + ; *UASp-α-Cat RNAi* / +	S6**AB**, 7**B**, S7**A**
♀ x ♂ *αTub-VP16* / *Ni::GFP* ; *Dl::mScarlet* / *UASp-w RNAi*	7**DC**, S7**B**
♀ x ♂ *αTub-VP16* / *Ni::GFP* ; *Dl::mScarlet* / *UASp-α-Cat RNAi*	7**DC**, S7**B**

Development • Supplementary information

Table S3. Primers used for qPCR

Primer	*Sequence*
α-Cat FWD 1	*ACCCGCTTGCTGATTTTAGCTG*
α-Cat REV 1	*TGCATTGCGTCCGAATTGCCTC*
α-Cat FWD 2	*GACGACTTTGACGAAGGAATTG*
α-Cat REV 2	*ATTGCATTCGGCCACAATTCTC*
RpL32 FWD	*CGGTTACGGATCGAACAAG*
RpL32 REV	*TCTGCATGAGCAGGACCTC*
RpII215 FWD	*GACTCGACTGGAATTGCACC*
RpII215 FWD	*TCTTCATCGGGATACTCGCC*

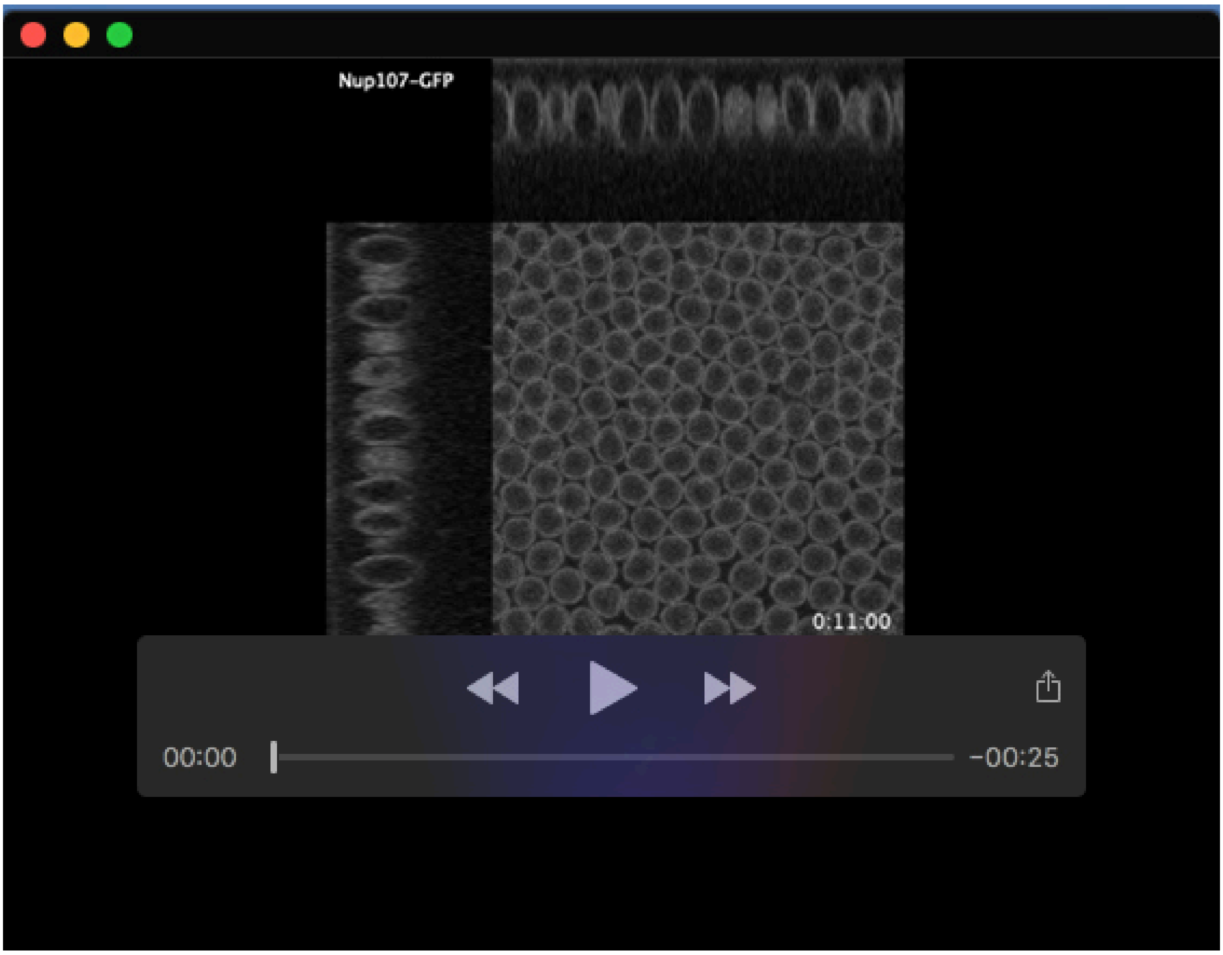

Movie 1. Changes in nuclear size and shape during nc14. Movie showing maximum projection of medial slices and orthogonal views of Nup107-GFP. 0.18 µm/px XY resolution and time resolution of 15s/frame. Anterior to the left; embryo imaged from the ventral side. Time indicates minutes from the beginning of nc14.

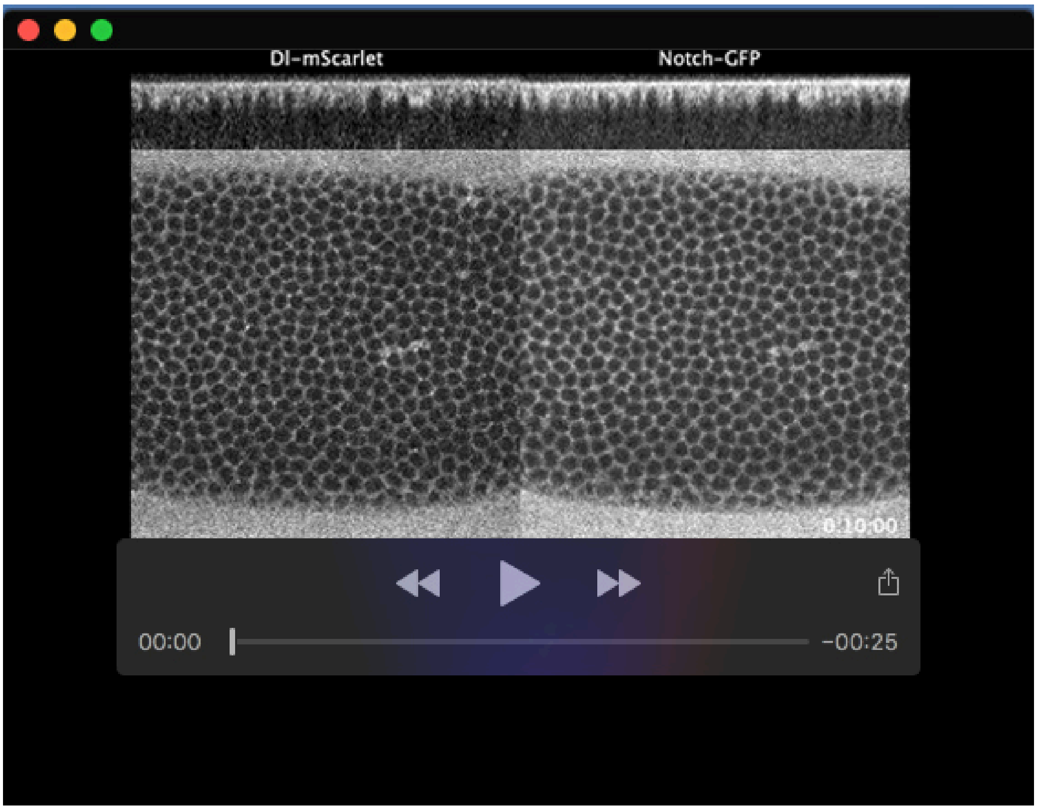

Movie 2. Localization of Notch and Delta during cellularization. Movie showing maximum projection of medial slices and orthogonal views of Dl-mScarlet (left) and Notch-GFP (right). 0.36 µm/px XY resolution and time resolution of 60s/frame. Anterior to the left; embryo imaged from the ventral side. Time indicates minutes from the beginning of nc14.

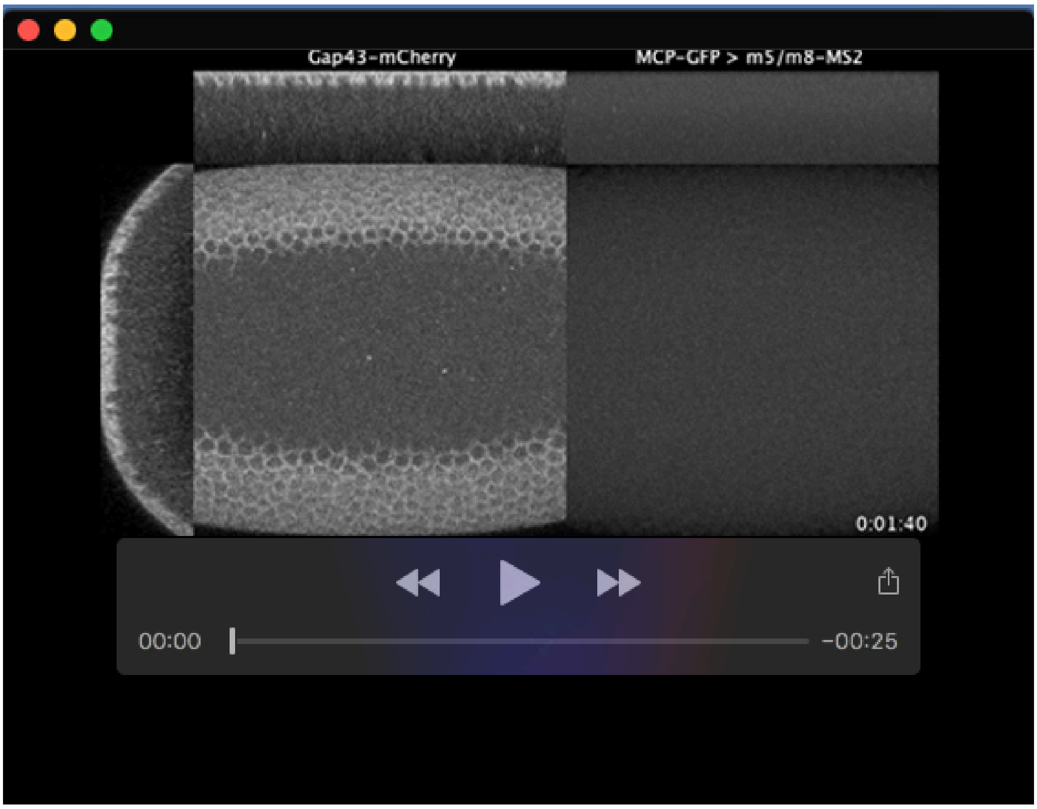

Movie 3. Expression of *m5/m8* starts during cellularization. Movie showing cellular izing membranes using the marker Gap43-mCherry (maximum intensity projection of medial slices and orthogonal views, left) and transcription from *m5/m8-MS2*[II] (max-imum intensity projection with maximum Y projection of the MCP-GFP channel, right). 0.36 µm/px XY resolution, 36x1µm slices and time resolution of 20s/frame. Anterior to the left; embryo imaged from the ventral side. Time indicates minutes from the beginning of nc14.

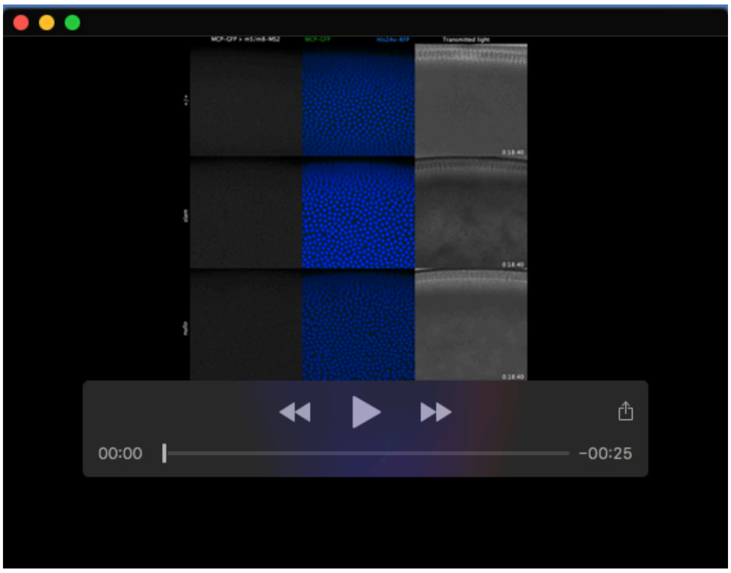

Movie 4. Expression of *m5/m8* in control, *slam* and *nullo* embryos. Movies showing MCP-GFP channel with transcription directed by *m5/m8*[IIII] (maximum intensity projection, left), His2Av-RFP channel in blue overlaid with MCP-GFP in green (maximum intensity projection, center) and transmitted light channel showing membrane growth (cross section, right) in control (top), *slam*$^{-/-}$ (middle) and *nullo*$^{-}$ (bottom) embryos. 0.36 µm/px XY resolution, 33x2µm slices and time resolution of 20s/frame. Anterior to the left; embryo imaged from the ventral side. Time indicates minutes from the beginning of nc14.

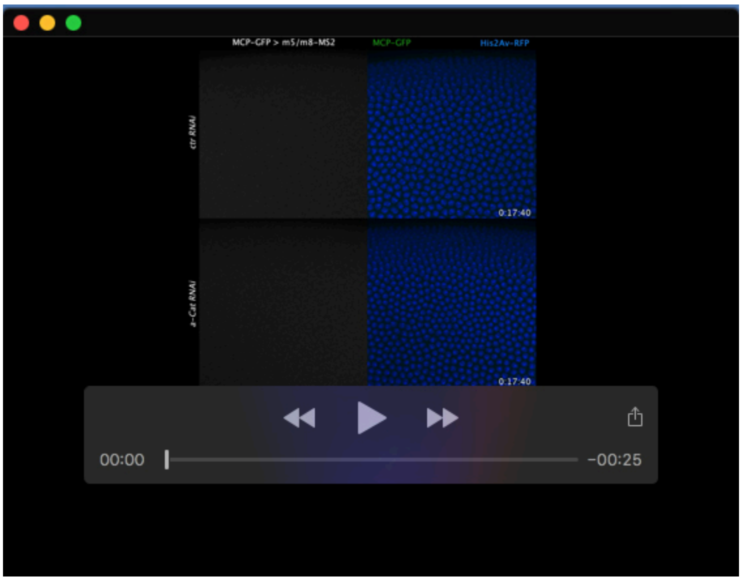

Movie 5. Expression of *m5/m8* in control and *α-Cat RNAi* embryos. Moviesshowing MCP-GFP channel with transcription directed by *m5/m8*[IIII] (maximum intensity projection, left) and His2Av-RFP channel in blue overlaid with MCP-GFP in green (maximum intensity projection, right) in control (top) and α-Cat depleted (bottom) embryos. 0.36 µm/px XY resolution, 32x1µm slices and time resolution of 20s/frame. Anterior to the left; embryo imaged from the ventral side. Time indicates minutes from the beginning of nc14.

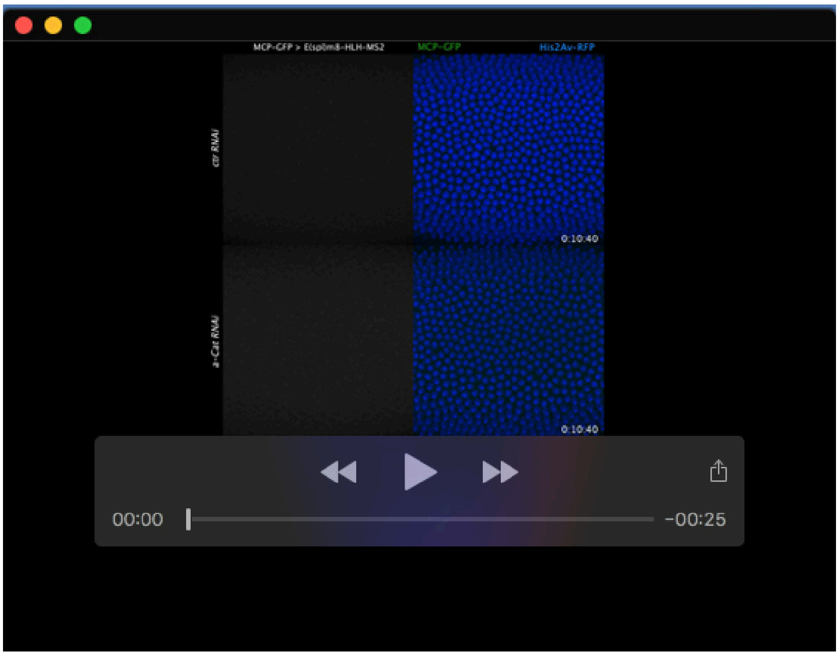

Movie 6. Expression of *E(spl)m8-HLH* in control and *α–Cat RNAi* embryos. Movies showing MCP-GFP channel with *E(spl)m8-HLH* transcription (maximum intensity projection, left) and His2Av-RFP channel in blue overlaid with MCP-GFP in green (maximum intensity projection, right) in control (top) and α-Cat depleted (bottom) embryos. 0.36 µm/px XY resolution, 32x1mm slices and time resolution of 20s/frame. Anterior to the left; embryo imaged from the ventral side. Time indicates minutes from the beginning of nc14.

Development • Supplementary information
