# Supplementary material for: Membrane architecture and adherens junctions contribute to strong Notch pathway activation
Source: Development. 2021 Oct 14;148(19):dev199831. doi: 10.1242/dev.199831 (PMC8543148; doi:10.1242/dev.199831)
Supplement: Reviewer comments [file develop-148-199831_review_history.pdf]

Membrane architecture and adherens junctions contribute to strong Notch pathway activation

Julia Falo-Sanjuan and Sarah J. Bray
DOI: 10.1242/dev.199831

Editor: Thomas Lecuit

Review timeline

Original submission:	27 May 2021
Editorial decision:	12 July 2021
First revision received:	23 July 2021
Accepted:	24 August 2021

Original submission

First decision letter

MS ID#: DEVELOP/2021/199831

MS TITLE: Membrane architecture and adherens junctions contribute to strong Notch pathway activation

AUTHORS: Julia Falo-Sanjuan and Sarah Bray

I have now received all the referees reports on the above manuscript, and have reached a decision. The referees' comments are appended below, or you can access them online: please go to BenchPress and click on the 'Manuscripts with Decisions' queue in the Author Area.

The overall evaluation is quite positive, though one of the reviewers is less enthusiastic about the work, and we would like to publish a revised manuscript in Development, provided that the referees' comments can be satisfactorily addressed. Please attend to all of the reviewers' comments in your revised manuscript and detail them in your point-by-point response. If you do not agree with any of their criticisms or suggestions explain clearly why this is so.

We are aware that you may currently be unable to access the lab to undertake experimental revisions. If it would be helpful, we encourage you to contact us to discuss your revision in greater detail. Please send us a point-by-point response indicating where you are able to address concerns raised (either experimentally or by changes to the text) and where you will not be able to do so within the normal timeframe of a revision. We will then provide further guidance. Please also note that we are happy to extend revision timeframes as necessary.

Reviewer 1

Advance summary and potential significance to field

This study examines when and how Notch signaling first occurs in the early fly embryo. Using a cutting-edge live imaging assay, the authors first show that the transcriptional onset of a Notch reporter is detected at mid-cellularization (Figs 1 and 2). This observation appears to be confirmatory since the authors previously used this assay to show that Notch-dependent transcription is first activated at mid-cellularization (30 min into cycle 14; Falo-Sanjuan et al.,

© 2021. Published by The Company of Biologists under the terms of the Creative Commons Attribution License (https://creativecommons.org/licenses/by/4.0/).

2019, Dev Cell). This observation also confirms earlier findings on the Notch-dependent transcription of the sim gene on fixed embryos.

The authors next tested whether disrupting nuclear lamina affects the onset of transcription and found that changes in nuclear morphology can be uncoupled from Notch-dependent transcription. The rationale of this experiment was, however, unclear since NICD is able to regulate transcription at the onset of cellularization, i.e. prior to changes in nuclear morphology. Why and how a change in nuclear morphology might affect the timing of Notch-dependent transcription needs to be better explained if this experiment has to be reported in the main text.

The authors then moved on to test whether the onset of Notch signaling is associated with the formation of lateral membranes during cellularization. This hypothesis makes obvious sense given that receptor activation requires interaction with its ligands at cell-cell contacts. In this developmental context, activation of Notch in the mesectoderm is known to require Delta endocytosis in the mesoderm, implying that cell-cell contacts along the mesoderm-ectoderm boundary are required for this signaling event. It is thus expected that the loss of lateral membranes in slam mutant embryos strongly reduces Notch-dependent transcription.

The authors then studied the onset of transcription at the single cell level relative to the progressive invagination of the lateral membrane and observed a correlation between the time of onset of transcription and the time when the membrane reaches the base of the nuclei. This raised the possibility that the onset of transcription is associated with dynamic changes in lateral membrane organization such as formation of Adherens Junctions (AJs). To test this, the authors used RNAi to deplete alpha-catenin. A decrease in the level of transcription was observed. By contrast the time of onset remained unchanged. Thus, AJs contribute to the strength of signaling but do not determine when Notch is activated. In order to address how alpha-catenin regulates Notch activity, the authors performed FRAP experiments. Results from these experiments indicate that alpha-catenin is somehow required to limit the mobility of Notch. Whether receptor mobility is causally linked to receptor activation was not further tested.

Comments for the author

While these data indicate that both membrane architecture and AJs contribute to strong Notch pathway activation (title), it is unclear how AJs positively regulate Notch receptor activation. Also, if membrane architecture (title) is meant to refer to the formation of cell-cell contacts at cellularization, then this conclusion is expected. Another limitation of this study is that the authors did not test whether the onset of Notch signaling simply depends on the onset of Delta endocytosis which might in turn depend on the onset of Neuralized expression. In summary, this paper is technically sound but fails to bring novel insights into how Notch is regulated in this developmental context.
Specific minor point - The authors claim that a delay in transcription onsets is observed in nullo mutant. This claim needs to be strengthened by performing a statistical analysis of the data shown in Fig 5E,F. Also, please explain the rationale for pooling the data from various genotypes to calculate R2 coefficients in Fig 5F, S4B and S5D. The data shown in Fig S5D were interpreted to state that there membrane length did not correlate with transcription onset time in nullo mutant embryos (p. 12). To support this claim, please compare the correlation coefficient values calculated for wild-type embryos and for nullo embryos. Since a single R2 value is provided, one wonders whether this value was calculated after pooling the data from the 3 genotypes. Please clarify.

Reviewer 2

Advance summary and potential significance to field

Notch signaling is initiated in the fly embryo in the ME during cellularization when lateral cell membranes and cell-cell adhesions first form. Falo-Sanjuan and Bray cleverly use this observation to identify elements of cellular architecture that contribute to Notch signaling. These authors conclude that the initiation of Notch signaling during cellularization is not dependent on nuclear shape changes but rather the formation of lateral cellular interfaces and adhesions. An interesting

© 2021. Published by The Company of Biologists under the terms of the Creative Commons Attribution License (https://creativecommons.org/licenses/by/4.0/).

advance here is the potential use of cellularization as a quantitative model to study cell architectural influences on Notch signaling.

Comments for the author

The comments below, if addressed, are unlikely to change the interpretation of the work, but would increase the rigor.

Concerns:
1) The authors are presenting a relatively short time for completion of cellularization, which may be achieved at some temperatures. However, the choice of Spider-GFP as a probe for furrow ingression also suggests that the timing is short simply because the authors cannot “see” the entire process. Spider-GFP signal becomes weaker at the end of cellularization, particularly at the basal ends of the furrows which must be visualized to follow the progression of cellularization. Perhaps the authors should (1) state the temperature at which their imaging was conducted; and/or (2) validate the timing of the cellularization process using a probe that better distinguishes the basal ends of furrows through the onset of gastrulation (e.g BL57144, BL57145 or other related Sqh-GFP stocks available throughout the community). As a related matter, the authors rarely discuss or show the furrow lengths that they observe, which could eventually be important if considering the length of lateral membrane that is juxtaposed at different times over the course cellularization.

2) Throughout, the authors show a strip of cells where Notch signaling is occurring and state that this is ME. Could this statement be validated with mesoderm or ME markers?

3) The kuk mutant phenotypes do not look consistent with prior published work on kuk (Brandt et al., 2006). Specifically, kuk mutants were previously reported to not undergo nuclear lengthening, and nuclei remained round; whereas these authors are showing nuclear morphologies that do not look very different than wild-type. Since no difference in Notch signaling is seen in these kuk mutants, it is critical to validate that these embryos do have kuk knockdown.

4) The treatment of the slam data is confusing and not completely substantiated, even by the authors’ own admission. It is reasonable to imagine that there are a range of slam mutant phenotypes. The most compelling result would be predicted to be seen with the strongest phenotype where no or very minimal lateral membranes form (Acharya et al., 2014). However, panel C shows that the slam-/- embryos that were analyzed show lateral membrane formation, just at a reduced rate. Yet, over time in the slam-/- embryos, the transcription does not increase even though furrow length is increasing, all be it slowly. In this dataset, the more compelling argument for the importance of increasing lateral membrane formation is seen in +/+ embryos where transcription is very low until 30 minutes into cellularization, after which the majority of lateral furrow would form and coincidentally transcription ramps up (also clearly seen in preceding experiments).

5) How does inclusion of the nullo data further the interpretation of the study? Is the idea that nullo allows dissection of the role of basal junctions versus apical junctions? This might be made a little more clear, and the lack of basal junctions in mutants shown.

6) FRAP is not a unit. What is depicted in Figure 7D? The plot should be appropriately labeled.

Minor concerns:
1) Notch and Delta appear to have an apical lateral bias (Figure 2), yet this is not emphasized. It would be interesting if the authors might address what if any significance they see this bias having on signaling.

2) It is not clear why the cell surface cartoons for the slam mutants are shown with slanting lateral membranes and the embryo surface shown with bent morphology.

3) How do the 2 slam-/- embryos analyzed in the supplemental materials relate to the 4 slam-/- embryos depicted in the main figure?

© 2021. Published by The Company of Biologists under the terms of the Creative Commons Attribution License (https://creativecommons.org/licenses/by/4.0/).

Reviewer 3

Advance summary and potential significance to field

This manuscript from Falo-Sanjuan and Bray explores whether Notch-Delta signaling is dependent on lateral plasma membranes and the potential transmembrane juxtaposition of N-Dl proteins in the Drosophila embryo. They use a nice MS2-based system to track the onset of transcription in response to N-Dl signaling, and show that this happens at mid-cellularization. They then explore whether this activation of N-Dl depends on transitions in nuclear shape and/or the growth or polarization of lateral membranes.
They use mutations to disrupt nuclear shape transitions (kugelkern) or various aspects of cellularization (slam, nullo, and a-catenin shRNA). Slam disruption appears to deeply affect the ability of N-Dl signaling to initiate, while nullo disruption appears to leave signaling mostly intact, albeit with some disruption of timing.
alpha-catenin disruption affects the overall levels of signaling. This suggests that some aspect of adhesion may affect N-Dl signaling, though it is unclear how this may occur. In general, I think this is an interesting study that should be of interest to the readers of Development. A bit greater care needs to be taken with some of the interpretation in the manuscript, and it seemed there were alternative explanations or caveats that were not mentioned at times, but after revision this would seem to be a good addition to Development.

Comments for the author

1) One major weakness of the study is that membrane invagination was not/could not be tracked at the same time as N-Dl signaling in the various disrupted backgrounds on a cell-by-cell basis. The expression data was roughly correlated with how well cellularization appeared to be happening by the somewhat crude measurements possible from trans-illumination (marking the general advancement of the cellularization front). This means that the authors were not able to directly correlate an individual nucleus' expression with the state of its lateral membranes/AJs/polarization. This produces a certain lack of clarity in these sections of the paper as Nullo (especially), Slam, and alpha-cat phenotypes can be variable, and there is therefore some guesswork on why certain nuclei behaved in specific ways. I suspect this is because the genetics to be able to mark membrane in these backgrounds is challenging, which is understandable, but a small acknowledgement of this limitation in the Results section would help to clarify this for the reader.

2) It seems like an alternative to the authors final hypothesis is that the juxtaposed membranes need to be closely enough apposed that N-Dl are at the proper distance for interaction, and that defects in AJ function may disrupt this close apposition (TEMs of alpha-cat cells often show this). Maybe not that different from some of the possible hypotheses that are mentioned, but I kept thinking that it was likely alterations in membrane geometries that were causing N-Dl defects rather than any direct effect (or effect on polarization), so it could be more explicitly stated.

3) I had a few questions from the Introduction:
- I believe all three of Notch, Delta, and Serrate are transmembrane proteins that are not freely diffusible in the extracellular space, is this correct? I wasn't sure about this for the all the available ligands and this is important information for the first Intro paragraph.
- I kept having the same question about the NICD experiment cited in the Intro. By expressing just the NICD, it would seem that it would be bypassing the time to express, activate, and transduce signaling through a variety of N-Dl steps, so expression of the downstream NICD doesn't seem equivalent to normal pathway temporal dynamics. I suspect the authors' interpretation of this result is correct, but could this be better justified? Or should this alternate be briefly mentioned as a possibility?
- The section on cellularization should be better cited. Postner and Wieschaus first described Nullo localization, for example.

4) When do apical junctions form? Would be important to know given the authors' hypothesis on their impact on N-Dl signaling.

© 2021. Published by The Company of Biologists under the terms of the Creative Commons Attribution License (https://creativecommons.org/licenses/by/4.0/).

5) Some of the labeling in the Figures is really small and hard to read. Please make it bigger.

6) Lines 158-160, seems like the authors can't rule out changes in nuclear pore function/organization, so a caveat to these lines is worth mentioning here.

7) Sorry if I missed it, but is there a reason why Delta localization is not looked at relative to the junctions in a similar fashion as for Notch? Should this be briefly mentioned?

8) Is the Notch:GFP used in the FRAP experiments endogenously labelled? Should be made clear.

Minor note:
a) Thought Fig. 1 was particularly nice, and the schematics throughout were a nice addition.
b) Line 170, "cases"? I suspect they are referring to embryos, but should be made clear. Also, "cellularization" is misspelled.
c) Line 171, make it clear that "no transcriptional activity" is not in general, but specific to the enhancers.
d) Fig. 4E "onset" of… transcription? Cellularization? Also, found 4C, confusing to look at at first, and it took effort to understand. I was looking for a Y-axis label and wondering what the tick marks correspond to –
how are nuclei grouped into these stages? Figure panel could be made more approachable.
e) Lines 272-274, this did not seem to necessarily follow from the prior sentence. Are the authors discussing in wild-type or alpha-catenin disrupted? Please clarify.
f) Fig. 7B, "NECD" label makes it seem that this is expression of the extracellular domain – suspect this isn't true, just referring to N antibodies that recognize an extracellular epitope? May want to add an "anti-" to label.

First revision

Author response to reviewers' comments

Below, the reviewer comments are in grey and our responses in black font.

Reviewer 1
Reviewer 1 Advance Summary and Potential Significance to Field:
This study examines when and how Notch signaling first occurs in the early fly embryo. Using a cutting-edge live imaging assay, the authors first show that the transcriptional onset of a Notch reporter is detected at mid-cellularization (Figs 1 and 2). This observation appears to be confirmatory since the authors previously used this assay to show that Notch-dependent transcription is first activated at mid-cellularization (30 min into cycle 14; Falo-Sanjuan et al., 2019, Dev Cell). This observation also confirms earlier findings on the Notch-dependent transcription of the sim gene on fixed embryos.

The authors next tested whether disrupting nuclear lamina affects the onset of transcription and found that changes in nuclear morphology can be uncoupled from Notch-dependent transcription. The rationale of this experiment was, however, unclear since NICD is able to regulate transcription at the onset of cellularization, i.e. prior to changes in nuclear morphology. Why and how a change in nuclear morphology might affect the timing of Notch-dependent transcription needs to be better explained if this experiment has to be reported in the main text.

The authors then moved on to test whether the onset of Notch signaling is associated with the formation of lateral membranes during cellularization. This hypothesis makes obvious sense given that receptor activation requires interaction with its ligands at cell-cell contacts. In this developmental context, activation of Notch in the mesectoderm is known to require Delta endocytosis in the mesoderm, implying that cell-cell contacts along the mesoderm-ectoderm boundary are required for this signaling event. It is thus expected that the loss of lateral membranes in slam mutant embryos strongly reduces Notch-dependent transcription.

© 2021. Published by The Company of Biologists under the terms of the Creative Commons Attribution License (https://creativecommons.org/licenses/by/4.0/).

The authors then studied the onset of transcription at the single cell level relative to the progressive invagination of the lateral membrane and observed a correlation between the time of onset of transcription and the time when the membrane reaches the base of the nuclei. This raised the possibility that the onset of transcription is associated with dynamic changes in lateral membrane organization such as formation of Adherens Junctions (AJs). To test this, the authors used RNAi to deplete alpha-catenin. A decrease in the level of transcription was observed. By contrast the time of onset remained unchanged. Thus, AJs contribute to the strength of signaling but do not determine when Notch is activated. In order to address how alpha-catenin regulates Notch activity, the authors performed FRAP experiments. Results from these experiments indicate that alpha-catenin is somehow required to limit the mobility of Notch. Whether receptor mobility is causally linked to receptor activation was not further tested.
We thank the reviewer for the comprehensive summary and are glad they find some of the approaches cutting edge. We have modified the text extensively to take on board the points raised.

Reviewer 1 Comments for the Author:
While these data indicate that both membrane architecture and AJs contribute to strong Notch pathway activation (title), it is unclear how AJs positively regulate Notch receptor activation. Also, if membrane architecture (title) is meant to refer to the formation of cell-cell contacts at cellularization, then this conclusion is expected.

We appreciate the points made by the reviewer. We note however that, although it may not be unexpected that cell-cell contacts and AJs are required for Notch activation, there is little data that demonstrate this point unequivocally and it is often difficult to tease apart the requirements *in vivo*. Indeed, as sumarized in the Introduction, there are several papers arguing that signalling occurs from intracellular membranes (cis-activation), and/or prior to the establishment of junctions. Because the lateral membranes are established *de novo* during cellularization in *Drosophila*, it offered a powerful system to investigate their role in signalling. The results confirm the expectations of the reviewer, but that does not make them less important.

Another limitation of this study is that the authors did not test whether the onset of Notch signaling simply depends on the onset of Delta endocytosis which might in turn depend on the onset of Neuralized expression. In summary, this paper is technically sound but fails to bring novel insights into how Notch is regulated in this developmental context.
We agree that the expression of Neuralized is very important for initiation of Notch signalling, a point that we had not made sufficiently clear in the original manuscript. We have modified the text in the Introduction (**page 3**) and in the Discussion (**page 12**) to stress the importance of Neuralized. However, if Neuralized was the sole factor limiting activation, we would not see a loss of signalling in *slam* mutants or any effects from depleting α-Catenin. We propose that the AJs, and the lateral membranes, are required for Neuralized to do its job.
In this context we note also that the author of one of the key papers describing the function of Neuralized at this time, has subsequently argued that signalling commences at the beginning of nc14 (Viswanathan et al. 2009). Our data do not support this and favour an integration of Neuralized with the other changes in cell morphology that occur in mid nc14, to precisely co-ordinate Notch signaling and the consequent transcription onset.

Specific minor point - The authors claim that a delay in transcription onsets is observed in nullo mutant. This claim needs to be strengthened by performing a statistical analysis of the data shown in Fig 5E,F.
We realise that we have presented some of the results in a confusing way, without stressing clearly the significance of the different observations. As the reviewer correctly points out, the transcriptional delay in the *nullo* mutants is not significant, and we have therefore moved these data to the supplementary. One of the main conclusions from the *nullo* experiments are that transcription can still initiate and that, on an embryo-by-embryo basis, transcription onset does not correlate with the length of the lateral membranes

Also, please explain the rationale for pooling the data from various genotypes to calculate R2 coefficients in Fig 5F, S4B and S5D. The data shown in Fig S5D were interpreted to state that there membrane length did not correlate with transcription onset time in nullo mutant embryos (p. 12). To support this claim, please compare the correlation coefficient values calculated for wild-type

© 2021. Published by The Company of Biologists under the terms of the Creative Commons Attribution License (https://creativecommons.org/licenses/by/4.0/).

embryos and for nullo embryos. Since a single R2 value is provided, one wonders whether this value was calculated after pooling the data from the 3 genotypes. Please clarify.
We thank the reviewer for raising this point. The strength of the analysis comes from comparing the variations in timing for membrane length and the variations in transcription onset for the individual embryos in an unbiased way (making no assumptions about genotype). The variations between wild-type embryos are very small, both in terms of membrane length and transcription starts, so they do not yield a good basis for comparison. We have clarified the approach in the text (**pages 8 and 10**) and, for transparency, we have added the analysis for the individual genotypes to the supplementary figures (**Figures S4 and S5**), which confirms there is a correlation for the *slam* progeny but not for *nullo*.

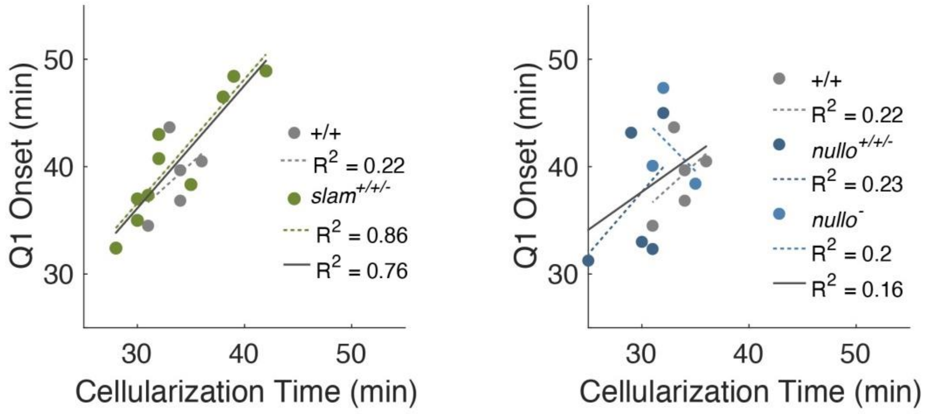

Reviewer 2
Advance Summary and Potential Significance to Field:
Notch signaling is initiated in the fly embryo in the ME during cellularization when lateral cell membranes and cell-cell adhesions first form. Falo-Sanjuan and Bray cleverly use this observation to identify elements of cellular architecture that contribute to Notch signaling. These authors conclude that the initiation of Notch signaling during cellularization is not dependent on nuclear shape changes, but rather the formation of lateral cellular interfaces and adhesions. An interesting advance here is the potential use of cellularization as a quantitative model to study cell architectural influences on Notch signaling.
We thank the reviewer for their positive comments.

Reviewer 2 Comments for the Author:
The comments below, if addressed, are unlikely to change the interpretation of the work, but would increase the rigor.

Concerns:
1) The authors are presenting a relatively short time for completion of cellularization, which may be achieved at some temperatures. However, the choice of Spider-GFP as a probe for furrow ingression also suggests that the timing is short simply because the authors cannot “see” the entire process.
Spider-GFP signal becomes weaker at the end of cellularization, particularly at the basal ends of the furrows which must be visualized to follow the progression of cellularization. Perhaps the authors should
(1) state the temperature at which their imaging was conducted; and/or (2) validate the timing of the cellularization process using a probe that better .distinguishes the basal ends of furrows through the onset of gastrulation (e.g BL57144, BL57145 or other related Sqh-GFP stocks available throughout the community). As a related matter, the authors rarely discuss or show the furrow lengths that they observe, which could eventually be important if considering the length of lateral membrane that is juxtaposed at different times over the course cellularization
We appreciate the concerns of the reviewer, and acknowledge that we were only able to use fairly coarse methods to measure the membranes due to imaging (and genetic) constraints. We also recognize that we cannot measure accurately the very late stages of cellularization, because of these limitations. However, as the events we are focusing on take place during the earlier period,

© 2021. Published by The Company of Biologists under the terms of the Creative Commons Attribution License (https://creativecommons.org/licenses/by/4.0/).

the latter does not impact on our interpretations. We have modified the text and adjusted the figures to make the limitations more explicit.

- We now explain limitations from the approaches used in the results (**pages 7- 8 and 9**).
- We have changed the final time--point in the diagram (**New figure 1E**) to >45, as we cannot be precise about the final end-point when cellularization is fully completed. The text has been similarly modified. We also adjusted the arrows in **Figure 1D and 1E** so they extend further, to prevent implying a strict end-point at the times indicated.
- We have added information about the temperature to the legend of Figure 1 and Methods.

2) Throughout, the authors show a strip of cells where Notch signaling is occurring and state that this is ME. Could this statement be validated with mesoderm or ME markers?

We apologise if we had not made clear that the *E(spl)m5/m8* enhancer we have been using was originally defined by the Levine lab, who validated its expression in the MSE very robustly (Zinzen et al. 2006). We have previously characterised this enhancer in conjunction with the MS2 system (Falo-Sanjuan et al. 2019). The references have been provided in the Introduction and we have now added them to the results section also (**Pages 3 and 5**).

3) The kuk mutant phenotypes do not look consistent with prior published work on kuk (Brandt et al., 2006). Specifically, kuk mutants were previously reported to not undergo nuclear lengthening, and nuclei remained round; whereas these authors are showing nuclear morphologies that do not look very different than wild-type. Since no difference in Notch signaling is seen in these kuk mutants, it is critical to validate that these embryos do have kuk knockdown.

We apologise that we had not made explicit the information about the allele of *kuk* that we were using. In order to produce homozygous embryos we have used *kuk[EY07696],* which reduces levels of Kuk by eliminating one of the *kuk* isoforms, and produces a milder phenotype than a *kuk* null (Pilot et al.
2006). We have now modified the text in the results to explain this point (**page 7**).

4) The treatment of the slam data is confusing and not completely substantiated, even by the authors' own admission. It is reasonable to imagine that there are a range of slam mutant phenotypes. The most compelling result would be predicted to be seen with the strongest phenotype where no or very minimal lateral membranes form (Acharya et al., 2014). However, panel C shows that the slam-/- embryos that were analyzed show lateral membrane formation, just at a reduced rate. Yet, over time in the slam-/- embryos, the transcription does not increase even though furrow length is increasing, all be it slowly. In this dataset, the more compelling argument for the importance of increasing lateral membrane formation is seen in +/+ embryos where transcription is very low until 30 minutes into cellularization, after which the majority of lateral furrow would form and coincidentally transcription ramps up (also clearly seen in preceding experiments).

We regret that our handling of the *slam* data was confusing and have rewritten this section extensively (**page 8**) and modified **Figure 4**, taking the reviewer's concerns into consideration.

- In the majority (3 / 4) of *slam* mutant embryos the growth of lateral membranes is arrested, consistent with previous results (Acharya *et al*. 2014). Panel C was misleading because it represented the timings from the 1 of 4 embryos that had a more sustained lateral membrane growth. No means should have been plotted. We have revised the panel (**New Figure 4C**) so there is a single dot in that column and added explanation in the legend and the text.
- In the heterozygous slam -/+ embryos, both lateral membrane growth and transcription is slowed, and the two are correlated. We have rewritten the text to clarify this point (**page 8**)
- We agree that one compelling argument for the importance of lateral membrane formation is the fact that transcription onset ramps up sharply at 30 minutes, when the apical portion of the lateral membrane will have formed. This observation is noted at the beginning of the results and was the starting point for this study. We have added a sentence at the start of the results to emphasise this more clearly (**page 5**). The key results from the *slam* experiment are that (i) arresting membrane development abrogates most activity (ii) slowing membrane growth results in a delay to onset timeless.

5) How does inclusion of the nullo data further the interpretation of the study? Is the idea that nullo allows dissection of the role of basal junctions versus apical junctions? This might be made a little

© 2021. Published by The Company of Biologists under the terms of the Creative Commons Attribution License (https://creativecommons.org/licenses/by/4.0/).

more clear, and the lack of basal junctions in mutants shown.

Nullo mutants further the interpretation in two ways. First, as the reviewer notes, the fact that *nullo* loss perturbs basal junctions, reported in detail by others, yet transcription still initiates, suggests that the BAJs are not essential for normal level of *m5/m8* transcription. Second, because there was much more variability in the onset time of transcription in the mutant embryos, we could exploit this, in a similar manner to the *slam* heterozygous embryos to investigate the relationship between membrane length and transcription start-times. In *nullo* mutant embryos, there was no correlation between the membrane growth and the onset time, unlike in *slam* conditions. This second finding is quite significant in the context of the models, suggesting the membrane dimension per se is not limiting. We have substantially rewritten and reorganized the results in this section (**pages 9-10**) and we hope this has clarified their relevance.

6) FRAP is not a unit. What is depicted in Figure 7D? The plot should be appropriately labeled.
We thank the reviewer for pointing this out and have now corrected the label on the axis to “Relative Fluorescence”.

Minor concerns:
1) Notch and Delta appear to have an apical lateral bias (Figure 2), yet this is not emphasized. It would be interesting if the authors might address what if any significance they see this bias having on signaling.
When comparing the distributions of spider-GFP and Notch or Delta, it appears that any apical bias may be more a consequence of the imaging, as similar bias is seen with this membrane marker (and others). Nevertheless, we have commented in the text on the possibility that there may be some apical bias, as suggested by the reviewer (**page 6**).

2) It is not clear why the cell surface cartoons for the slam mutants are shown with slanting lateral membranes and the embryo surface shown with bent morphology.
The figures have been modified as suggested (**Figure 3, 5 and S6**).

3) How do the 2 slam-/- embryos analyzed in the supplemental materials relate to the 4 slam-/- embryos depicted in the main figure?
The two embryos in the supplementary are from a genetic combination with Dl-mScarlet. Those in the main text are from a combination with the MS2 reporters, nos-MCP-GFP and His2Av-RFP. This has been stated explicitly in the l**egend of Figure S4**.

Reviewer 3
Advance Summary and Potential Significance to Field:
This manuscript from Falo-Sanjuan and Bray explores whether Notch-Delta signaling is dependent on lateral plasma membranes and the potential transmembrane juxtaposition of N-Dl proteins in the Drosophila embryo. They use a nice MS2-based system to track the onset of transcription in response to N-Dl signaling, and show that this happens at mid-cellularization. They then explore whether this activation of N-Dl depends on transitions in nuclear shape and/or the growth or polarization of lateral membranes. They use mutations to disrupt nuclear shape transitions (kugelkern) or various aspects of cellularization (slam, nullo, and a-catenin shRNA). Slam disruption appears to deeply affect the ability of N-Dl signaling to initiate, while nullo disruption appears to leave signaling mostly intact, albeit with some disruption of timing. alpha-catenin disruption affects the overall levels of signaling. This suggests that some aspect of adhesion may affect N-Dl signaling, though it is unclear how this may occur. In general, I think this is an interesting study that should be of interest to the readers of Development. A bit greater care needs to be taken with some of the interpretation in the manuscript, and it seemed there were alternative explanations or caveats that were not mentioned at times, but after revision this would seem to be a good addition to Development.

We are glad the reviewer finds the study of general interest. In revising the manuscript we have endeavored to be more cautious and to make clear the caveats.

Reviewer 3 Comments for the Author:
1) One major weakness of the study is that membrane invagination was not/could not be tracked at the same time as N-Dl signaling in the various disrupted backgrounds on a cell-by-cell basis. The

© 2021. Published by The Company of Biologists under the terms of the Creative Commons Attribution License (https://creativecommons.org/licenses/by/4.0/).

expression data was roughly correlated with how well cellularization appeared to be happening by the somewhat crude measurements possible from trans-illumination (marking the general advancement of the cellularization front). This means that the authors were not able to directly correlate an individual nucleus' expression with the state of its lateral membranes/AJs/polarization. This produces a certain lack of clarity in these sections of the paper as Nullo (especially), Slam, and alpha-cat phenotypes can be variable, and there is therefore some guesswork on why certain nuclei behaved in specific ways. I suspect this is because the genetics to be able to mark membrane in these backgrounds is challenging, which is understandable, but a small acknowledgement of this limitation in the Results section would help to clarify this for the reader.
We appreciate the concerns of the reviewer, and acknowledge that we were only able to use fairly coarse methods to measure the membranes due to imaging (and genetic) constraints. We agree that, because of the genetic constraints, which mean that we have not been able to combine the MS2 system with a membrane marker and the *nullo* allele, we can only hypothesize why certain nuclei exhibit altered activity in *nullo* mutants. We have modified the text to make the limitations more explicit (**pages 7-8 and 9**) and to add caution to the interpretation.

2) It seems like an alternative to the authors final hypothesis is that the juxtaposed membranes need to be closely enough apposed that N-Dl are at the proper distance for interaction, and that defects in AJ function may disrupt this close apposition (TEMs of alpha-cat cells often show this). Maybe not that different from some of the possible hypotheses that are mentioned, but I kept thinking that it was likely alterations in membrane geometries that were causing N-Dl defects rather than any direct effect (or effect on polarization), so it could be more explicitly stated.
We thank the reviewer for reminding us about this possibility. We have added statements in the discussion to include this possibility (**page 14**).

3) I had a few questions from the Introduction:
- I believe all three of Notch, Delta, and Serrate are transmembrane proteins that are not freely diffusible in the extracellular space, is this correct? I wasn't sure about this for the all the available ligands, and this is important information for the first Intro paragraph.
We have expanded the text in the first paragraph of the introduction to clarify that, except for some exceptions in *C.elegans*, the ligands and the receptor are transmembrane proteins.

- I kept having the same question about the NICD experiment cited in the Intro. By expressing just the NICD, it would seem that it would be bypassing the time to express, activate, and transduce signaling through a variety of N-Dl steps, so expression of the downstream NICD doesn't seem equivalent to normal pathway temporal dynamics. I suspect the authors' interpretation of this result is correct, but could this be better justified? Or should this alternate be briefly mentioned as a possibility?
We agree with the reviewer that expressing NICD is not comparable to the normal pathway dynamics. Indeed, that is key to the interpretations and relevance of those experiments. We apologise if this was opaque. We have now clarified that ectopic NICD was produced from the beginning of nc14 and bypasses the need for ligand activation and membrane trafficking (**Page 3**). We comment on this experiment to justify that the chromatin landscape and essential transcription factors are not limiting at this stage, rather the limitations must affect processes that are required to activate and transduce signaling,

- The section on cellularization should be better cited. Postner and W ieschaus first described Nullo localization, for example.
We thank the reviewer for highlighting this and have added relevant references.

.4) When do apical junctions form? Would be important to know given the authors' hypothesis on their impact on N-Dl signaling
We have added information about when the AAJs form in the results (**Page 10**).

5) Some of the labeling in the Figures is really small and hard to read. Please make it bigger. The size of the font has been adjusted in the Figures, in those cases where it was very small.

6) Lines 158-160, seems like the authors can't rule out changes in nuclear pore function/organization, so a caveat to these lines is worth mentioning here.

© 2021. Published by The Company of Biologists under the terms of the Creative Commons Attribution License (https://creativecommons.org/licenses/by/4.0/).

We appreciate the point made by the reviewer and have added a caveat as suggested (**Page 7**).

7) Sorry if I missed it, but is there a reason why Delta localization is not looked at relative to the junctions in a similar fashion as for Notch? Should this be briefly mentioned?
We could not perform the same high level analysis of Delta localization, as for Notch, for technical reasons. The antibody staining did not have good enough signal to noise to produce SIM images and the Dl-mScralet bleached too fast in the FRAP. These points were mentioned in the methods and have been added in the main text/legends . We have realized however that it would be helpful to include data from live imaging of Dl-mScarlet in the alpha-Catenin knock down, demonstrating that there are no gross changes. These data have now been added to **new Figure S7** and a comment made in the results (**page 11**).

8) Is the Notch:GFP used in the FRAP experiments endogenously labelled? Should be made clear.
The Notch-GFP is carried on a BAC rescue construct, expressed at endogenous levels. This has been noted in the main text, methods and figure legend (**Figure 7 and pages 6 and 11**).

Minor note:
a) Thought Fig. 1 was particularly nice, and the schematics throughout were a nice addition.
Thank you, we are glad the schematics are helpful.

b) Line 170, "cases"? I suspect they are referring to embryos, but should be made clear. Also, "cellularization" is misspelled.
Thank you for the suggestion and for noting the typo, both have been corrected.

c) Line 171, make it clear that "no transcriptional activity" is not in general, but specific to the enhancers.
This has been checked and clarified through-out.

d) Fig. 4E "onset" of… transcription? Cellularization? Also, found 4C, confusing to look at at first, and it took effort to understand. I was looking for a Y-axis label and wondering what the tick marks correspond to – how are nuclei grouped into these stages? Figure panel could be made more approachable.
We have modified **Figure 4** to address the concerns of the reviewer, stating that 4E is time from transcription onset, and removing the confusing tick marks.

e) Lines 272-274, this did not seem to necessarily follow from the prior sentence. Are the authors discussing in wild-type or alpha-catenin disrupted? Please clarify.
We have clarified that we are discussing the effects on wild-type embryos, which are inferred from the alpha-Catenin knock-down results (**page 12**).

f) Fig. 7B, "NECD" label makes it seem that this is expression of the extracellular domain – suspect this isn't true, just referring to N antibodies that recognize an extracellular epitope? May want to add an "anti-" to label.
Thank you for the helpful suggestion, this has been done.

<u>Second decision letter</u>

MS ID#: DEVELOP/2021/199831

MS TITLE: Membrane architecture and adherens junctions contribute to strong Notch pathway activation

AUTHORS: Julia Falo-Sanjuan and Sarah Bray
ARTICLE TYPE: Research Article

© 2021. Published by The Company of Biologists under the terms of the Creative Commons Attribution License (https://creativecommons.org/licenses/by/4.0/).

I have looked carefully at the response from the 3 reviewers. Although one of the reviewers questions the novelty of the work, in my opinion, the evidence provided here establish on firmer grounds a conclusion which indeed has been proposed earlier thereby confirming previous results. The confirmatory aspect of the work and the quality of the data, praised by all reviewers, lead me to accept the manuscript for publication in Development, pending our standard ethics checks.

Reviewer 1

Advance summary and potential significance to field

The authors have addressed the two minor points raised in my review. These revisions do not, however, change my overall evaluation (lack of novel insights). The notion that cell-cell contacts are required for Notch signaling is well demonstrated experimentally and I believe that few would dispute this notion. In their rebuttal, the authors highlight two studies proposing that Notch may also be activated intracellularly. However, the data reported herein do not appear particularly strong so that this possibility seems to remain speculative at this stage. Thus, the conclusion that cell-cell contacts is important for Notch signaling may not warrant publication at Development. Otherwise, how AJs modulate Notch activity in this specific context remains unknown and it is therefore unclear whether this conclusion is of general significance. Considering that signaling starts with the onset of Delta endocytosis, one may wonder whether and how low a-catenin levels affects the endocytosis of Delta in the fly embryo. This remains to be examined.

Comments for the author

-

Reviewer 2

Advance summary and potential significance to field

See prior review.

Comments for the author

The reviewer concerns have been adequately addressed.

Reviewer 3

Advance summary and potential significance to field

The authors have responded appropriately to reviewer comments. The revised manuscript is nicely done, and it should make a good addition to Development. Congratulations on the nice study!

Comments for the author

The authors have responded appropriately to reviewer comments. The revised manuscript is nicely done, and it should make a good addition to Development. Congratulations on the nice study!

© 2021. Published by The Company of Biologists under the terms of the Creative Commons Attribution License (https://creativecommons.org/licenses/by/4.0/).
